# Smartphone-Based Hand Function Assessment: Systematic Review

**DOI:** 10.2196/51564

**Published:** 2024-09-16

**Authors:** Yan Fu, Yuxin Zhang, Bing Ye, Jessica Babineau, Yan Zhao, Zhengke Gao, Alex Mihailidis

**Affiliations:** 1 School of Mechanical Science and Engineering Huazhong University of Science and Technology Wuhan China; 2 KITE - Toronto Rehabilitation Institute University Health Network Toronto, ON Canada; 3 Department of Occupational Science and Occupational Therapy University of Toronto Toronto, ON Canada; 4 Library and Information Services University Health Network Toronto, ON Canada; 5 Department of Rehabilitation Medicine Hubei Province Academy of Traditional Chinese Medicine Hubei Provincial Hospital of Traditional Chinese Medicine Wuhan China

**Keywords:** hand function assessment, smartphone-based sensing, rehabilitation, digital health, mobile health, mHealth, mobile phone

## Abstract

**Background:**

Hand function assessment heavily relies on specific task scenarios, making it challenging to ensure validity and reliability. In addition, the wide range of assessment tools, limited and expensive data recording, and analysis systems further aggravate the issue. However, smartphones provide a promising opportunity to address these challenges. Thus, the built-in, high-efficiency sensors in smartphones can be used as effective tools for hand function assessment.

**Objective:**

This review aims to evaluate existing studies on hand function evaluation using smartphones.

**Methods:**

An information specialist searched 8 databases on June 8, 2023. The search criteria included two major concepts: (1) smartphone or mobile phone or mHealth and (2) hand function or function assessment. Searches were limited to human studies in the English language and excluded conference proceedings and trial register records. Two reviewers independently screened all studies, with a third reviewer involved in resolving discrepancies. The included studies were rated according to the Mixed Methods Appraisal Tool. One reviewer extracted data on publication, demographics, hand function types, sensors used for hand function assessment, and statistical or machine learning (ML) methods. Accuracy was checked by another reviewer. The data were synthesized and tabulated based on each of the research questions.

**Results:**

In total, 46 studies were included. Overall, 11 types of hand dysfunction–related problems were identified, such as Parkinson disease, wrist injury, stroke, and hand injury, and 6 types of hand dysfunctions were found, namely an abnormal range of motion, tremors, bradykinesia, the decline of fine motor skills, hypokinesia, and nonspecific dysfunction related to hand arthritis. Among all built-in smartphone sensors, the accelerometer was the most used, followed by the smartphone camera. Most studies used statistical methods for data processing, whereas ML algorithms were applied for disease detection, disease severity evaluation, disease prediction, and feature aggregation.

**Conclusions:**

This systematic review highlights the potential of smartphone-based hand function assessment. The review suggests that a smartphone is a promising tool for hand function evaluation. ML is a conducive method to classify levels of hand dysfunction. Future research could (1) explore a gold standard for smartphone-based hand function assessment and (2) take advantage of smartphones’ multiple built-in sensors to assess hand function comprehensively, focus on developing ML methods for processing collected smartphone data, and focus on real-time assessment during rehabilitation training. The limitations of the research are 2-fold. First, the nascent nature of smartphone-based hand function assessment led to limited relevant literature, affecting the evidence’s completeness and comprehensiveness. This can hinder supporting viewpoints and drawing conclusions. Second, literature quality varies due to the exploratory nature of the topic, with potential inconsistencies and a lack of high-quality reference studies and meta-analyses.

## Introduction

### Background

Hand function assessment is crucial in determining the extent of functional loss in patients and the outcome of surgical and rehabilitative procedures. Subtle changes in hand function could be a good predictor for the early detection of certain neuromuscular degeneration diseases, such as Parkinson disease (PD), which could help take preventive measures to reduce the severity of the illness [1]. However, most current hand function assessments are conducted in a clinical context with the intensive involvement of rehabilitation professionals. Clinical evaluation requires frequent visits and long-duration treatment sessions [2]. Hand function is usually assessed using standard questionnaires, such as the Michigan Hand Outcome Questionnaire and Disability of the Arm, Shoulder, and Hand Index [3]. These measurements are subjective and could result in different assessment results across different test scenarios and medical professionals [4]. Clinical outcomes based on a rating scale are often insensitive to subtle hand function changes and do not support the provision of timely feedback [5]. As such, a hand assessment tool that can overcome the clinical assessment drawbacks of inconvenience, high cost, and imprecision [1,5] and automatically evaluate hand function over time would benefit patients.

Smartphones are equipped with advanced technologies, such as touchscreens, accelerometers, and gyroscopes, which can be used for measuring and evaluating hand function [6]. The application of smartphones in clinical hand dysfunction assessments can exploit built-in sensors (such as accelerometers and gyroscopes) to collect real-time hand movement data with convenience and at low cost [7]. Smartphones can precisely monitor and analyze a patient’s hand condition for dysfunction assessment using machine learning (ML) and artificial intelligence algorithms [8]. Moreover, the smartphone-based hand dysfunction assessment can be designed according to clinical criteria to improve the system’s reliability and validity [9-11]. Despite recent advances in smartphone-based hand function assessment [12,13], no systematic reviews have been conducted to provide a holistic perspective on how smartphones can be applied to hand function assessment.

Although other technologies, such as wrist-worn or finger-worn sensors, smartwatches, and specialized keyboards, also show potential for automated hand function assessment, they typically focus on simple physiological data collection with limited data processing capabilities and display of basic information [14-16]. However, smartphones offer more extensive data acquisition, accurate data processing, and richer data display options, providing a more comprehensive technological solution [17,18]. Moreover, considering the widespread availability and user-friendly nature of smartphones [19], directing research efforts toward smartphone-centric studies can enhance innovation and application possibilities. This approach not only aligns with the current prevalence of smartphones but also extends a broader scope for future technology transfer and development specific to hand function assessment. Therefore, focusing on smartphone research can lead to more innovation and application possibilities, offering a broader scope for future technology transfer and development. As such, the main goal of this review was to synthesize the present ways in which smartphones are applied in hand function assessment and the extent to which hand function evaluation is achieved using smartphones. It aimed to explore the system development guidelines for the future application of smartphones in hand function assessment.

### Research Questions

The research questions (RQs) were as follows: (1) What types of hand dysfunctions are studied, and what assessment inventory tools are used? (2) How are smartphones applied in clinical practice in hand function assessment? (3) What sensors are integrated into smartphones to collect hand function data? (4) What statistics or ML algorithms are used for hand function assessment?

## Methods

This systematic review is reported according to PRISMA (Preferred Reporting Items for Systematic Reviews and Meta-Analyses) guidelines (Multimedia Appendix 1).

### Information Sources and Search Strategy

An information specialist (JB) developed and executed a comprehensive search strategy. The following electronic databases were searched: MEDLINE(R) ALL (Ovid), Embase and Embase Classic (Ovid), CENTRAL (Ovid), Scopus, Compendex (Engineering Village), INSPEC (Engineering Village), IEEE Xplore, and ACM Digital Library. The search strategy was first developed in MEDLINE ALL (Ovid) in consultation with the research team. Search terms were also sourced from a previously published review [20]. The search strategy was then adapted into other databases.

Search strategies included the use of text words and subject headings related to two major concepts: (1) smartphone or mobile phone or mHealth and (2) hand function or function assessment. Searches were limited to English-language papers. When possible, searches were also limited to human studies and excluded conference proceedings and trial register records. No date limits were applied. All searches were conducted on June 8, 2023. The complete search strategies for each database are provided in Multimedia Appendix 2.

### Study Selection

The studies were imported into Covidence (Veritas Health Innovation) after eliminating duplicates using EndNote (Clarivate). Title and abstract screening and full-text screening were completed by 2 researchers (YZ and YF) independently based on the same inclusion and exclusion criteria. Any disagreement was first discussed and solved by the 2 researchers. Otherwise, a third researcher (BY) was involved to ensure that an agreement was reached.

Neurocognition is evaluated as an independent criterion in clinical hand assessments [21]. Therefore, neurocognitive studies were excluded from this review to focus specifically on aspects related to hand motor control and dysfunction. Although cognitive functions play a significant role in hand motor control, the primary aim of this review was to narrow its scope and focus on the specific factors directly related to the mechanics and dysfunction of the hand, with particular focus on methods and techniques for using smartphones in assessment. Neurocognitive research often involves specialized equipment and methods, for example, neuroimaging techniques such as functional magnetic resonance imaging or electroencephalogram, which may not be practical for assessing hand function in smartphone-related contexts.

After the screening stage, the research quality of selected studies was evaluated using the Mixed Methods Appraisal Tool, a tool designed for the systematic mixed research review evaluation phase [22]. The quality assessment was completed by one researcher and checked by another researcher. A conflict that arose regarding the assessment was discussed between the 2 researchers, and an agreement was reached.

The inclusion and exclusion criteria used for the screening process are presented in Textbox 1.

**The inclusion and exclusion criteria used for the screening process.**

**Inclusion criteria**

- Technology: using smartphone sensors
- Study focus: hand function screening, including hand movement assessment and hand performance measurement
- Clinical assessment: measurement of motor function–related criteria, such as grip strength, posture, and degree of freedom
- Study design: peer-reviewed academic studies
- Language: English

Population: human participants

**Exclusion criteria**

- Technology: not using a smartphone for hand function assessment
- Study focus: health management and neurocognitive studies
- Clinical assessment: qualitative, non–peer-reviewed, and nonacademic studies
- Study design: systematic reviews, literature reviews, case reports, and letters
- Language: non-English

Population: nonhuman participants

## Results

### Overview

**Figure 1:**
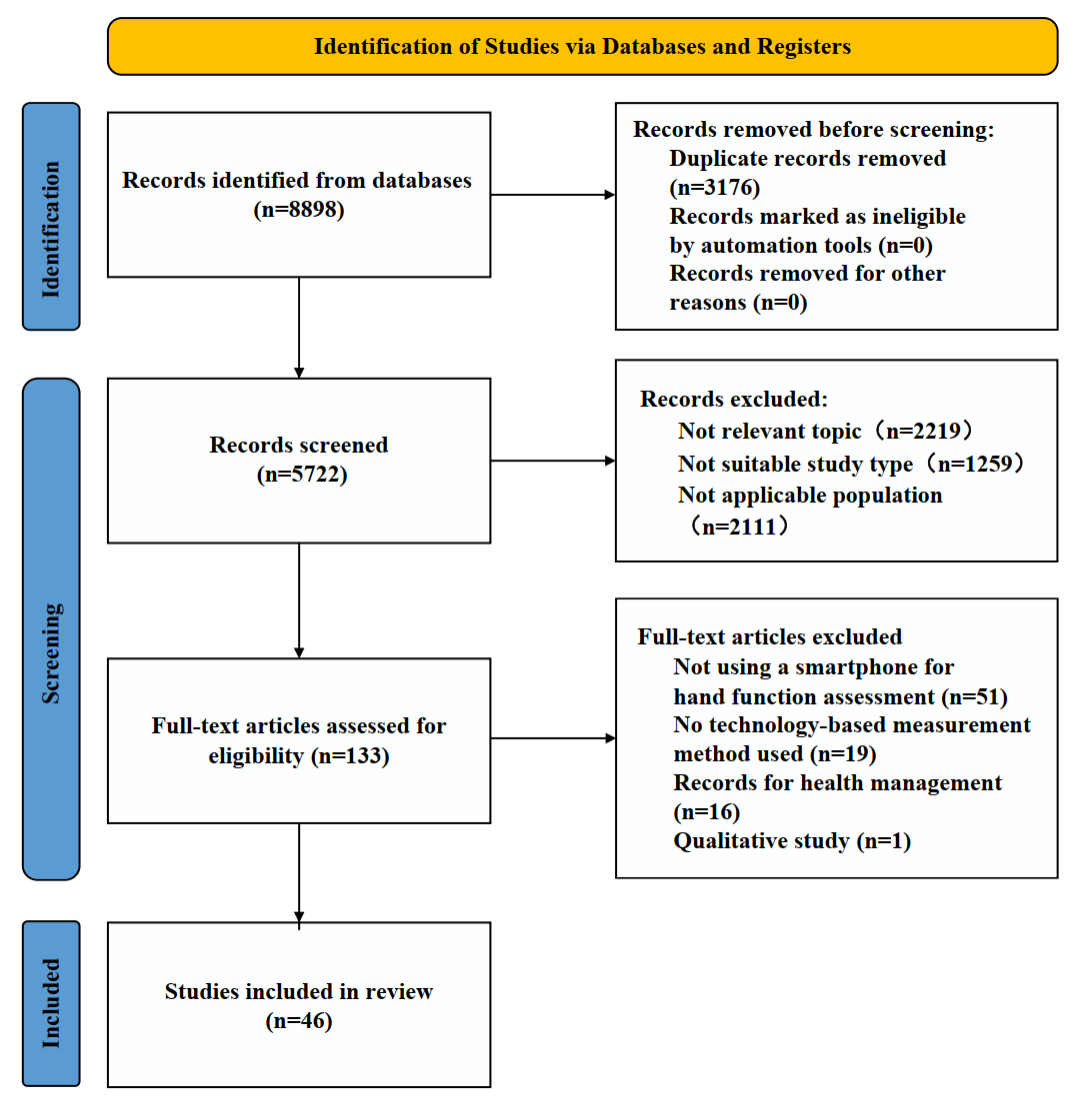
PRISMA (Preferred Reporting Items for Systematic Reviews and Meta-Analyses) flow diagram illustrating the screening process for papers included in this study.

A total of 8898 records were retrieved from the search. After removing duplicates, 64.31% (5722/8898) of the records were filtered at the title and abstract screening stage. After title and abstract screening, 97.68% (5589/5722) of the records were removed. The remaining 2.32% (133/5722) of the records underwent full-text screening. A total of 46 studies were included after both screening stages and included in the final review. Figure 1 presents the PRISMA [23] flow diagram. Multimedia Appendix 3 [6,9-11,24-58] details the results of the evaluation of included studies based on the Mixed Methods Appraisal Tool. All 46 studies were published after 2012, and 67% (n=31) of them were published between 2017 and 2023.

### Study Characteristics

Of the 46 studies, 14 (30%) recruited participants with hand dysfunction, 7 (15%) included only healthy participants, and 23 (50%) recruited both types of participants (Table 1). The summarized smartphone specification is shown in Table 2. The age range was 21 to 91 years for patients with hand dysfunction and 17 to 81 years for healthy participants; the sample size varied from 1 to 1815.

**Table 1:** Characteristics of the studies (n=46).

Characteristic			References
**Participants**			
	Patients only	[9,24-36]	
	Healthy participants only	[37-41,59,60]	
	Patients and healthy participants	[6,10,11,29,42-55,61-65]	
	—^a^	[56,57]	
**Sex**			
	Male only	[25,37]	
	Female only	[58]	
	Male and female	[6,9-11,26-28,30,31,33-35,39,40,42,43,45,48,49,52,53,55,56,59-65]	
	—	[24,29,32,36,38,41,44,46,47,50,51,54,57]	
**Study design**			
	Quantitative descriptive study	[9,24-29,31-42,45,46,51,56,57,59,60]	
	Observation study	[43,44,47,48,52,62,64,66]	
	Nonrandomized study	[6,10,11,25,30,38,43,44,47-50,52-55,58,61-65]	
	Case-control study	[58]	
**Study duration**			
	0-4 minutes	[10,28,29,31,39,58,64]	
	10 minutes	[59]	
	1.5 hours	[48]	
	10 hours	[61]	
	1-4 weeks	[9,26,42,52]	
	6-12 weeks	[51,63,66]	
	—	[11,24,25,27,30,32-38,40,41,43-47,49,50,52-57,60,62,65]	
**Sample size distribution**			
	0-32	[10,24-26,31-33,37-39,42,45,52,57,58,60,64]	
	33-64	[27,28,30,35,40,41,46,47,49,53,55,62,65]	
	65-95	[11,34,51,61,66]	
	96-126	[9,44,48,56]	
	127-189	[43,59]	
	190-220	—	
	221-252	[29]	
	253-598	—	
	599-629	[63]	
	630-1851	[36,50,54]	

^a^Not applicable.

**Table 2:** Summary of smartphone specification.

Study, year	Processing power	Operating system	Smartphone type	Sensor sampling rate	Camera resolution
Matera et al [26], 2016	—^a^	Android	Nuans Neo Reloaded and HUAWEI GR5	—	—
Miyake et al [24], 2020	1.2 GHz dual-core processor	—	—	Accelerometer (range +2 to –2 g, 100 Hz)	—
García-Magariño et al [42], 2016	—	Android	Samsung Galaxy Trend Plus	—	—
Bercht et al [25], 2012	—	iOS and Android 4.4.2	iPhone 4S，Samsung Galaxy S4, and Google Nexus 5	—	—
Janarthanan et al [39], 2020	—	Android	LG Optimus G smartphone	—	—
Pan et al [28], 2015	—	iOS	iPhone	Accelerometer (100 Hz)	—
Orozco-Arroyave et al [61], 2020	—	Android	Android smartphone	Accelerometer (100 Hz)	—
Sarwat et al [32], 2021	—	—	—	—	—
Kostikis et al [10], 2015	—	Android	—	Accelerometer and gyroscope (20 Hz)	—
Lee et al [43], 2016	—	Android	Galaxy S3 mini and Android phone	—	—
Lipsmeier et al [6], 2018	—	Android	Tablet	Accelerometer and gyroscope (+66.6 to –10 Hz), magnetometer (+66.6 to –7 Hz), and microphone (44.1 kHz)	—
Sandison et al [45], 2020	—	—	—	—	—
Halic et al [46], 2014	—	iOS	iPhone 5	—	—
Koyama et al [30], 2021	—	—	—	—	—
Chén et al [51], 2020	—	iOS	iPhone 4	—	—
Arroyo-Gallego et al [62], 2017	—	Android 7.0	Huawei P9 Plus	Custom screen keyboard (1.2 GHz)	—
Pratap et al [63], 2020	—	—	Huawei Mate 9 Pro smartphone	—	—
Waddell et al [64], 2021	—	—	—	App touchscreen, accelerometer, and gyroscope (50 Hz)	—
Mousavi et al [56], 2020	—	Android 4.0	—	Mobile accelerometer software (100 Hz)	—
Lee et al [55], 2016	—	—	—	—	—
Hidayat et al [58], 2015	—	—	Huawei P10 Lite	—	—
Wang et al [37], 2016	—	—	—	—	—
Lee et al [38], 2018	—	Android	—	—	—
Iakovakis et al [44], 2019	—	iOS	iPhone XS Max	—	—
Modest et al [47], 2019	—	iOS	iPhone XS Max	—	—
Lendner et al [59], 2019	—	iOS	iPhone	—	—
Tian et al [48], 2019	—	Android	Samsung Galaxy S3 Mini	—	—
Ge et al [27], 2020	—	Android	—	—	20 million pixels
Lee et al [9], 2016	—	Android	LG Optimus S smartphone	—	—
Reed et al [29], 2022	—	Android 5.0	Motorola Moto G II	—	—
Williams et al [31], 2021	—	Android 2.2	HTC Desire smartphone	—	60 frames per second and 1920×1080–pixel resolution
Gu et al [49], 2022	—	Android	Sony Xperia	—	Image resolution: 1980×1080 pixels
Gu et al [60], 2023	—	iOS	iPhone 5 or a newer device	—	Image resolution: 1980×1081 pixels
Prince et al [50], 2018	—	Android	—	—	—
Arora et al [52], 2015	—	—	—	—	—
Kassavetis et al [33], 2015	—	—	Huawei Mate 9 Pro	Smartphone accelerometers (50 Hz)	—
Ienaga et al [41], 2022	—	—	—	—	—
Espinoza et al [34], 2016	—	iOS	iPhone SE	—	—
Chén et al [51], 2020	—	—	—	—	20 million pixels
Surangsrirat et al [36], 2022	—	iOS	iPhone	—	—
Williams et al [53], 2020	—	iOS and Android	iPhone 11 Pro Max	—	60 frames per second, 1920×1080 pixels
Williams et al [11], 2020	—	Android	—	—	—
Prince and de Vos [54], 2018	—	Android	—	Smartphone app, screen, and accelerometer (100 Hz)	—
Santos et al [65], 2017	—	Ios	iPhone 5	—	—
Porkodi et al [40], 2023	—	Android	—	—	2400×1080–pixels and 64 megapixel f/1.89
Akhbardeh et al [57], 2015	—	—	Sony Xperia Z1	—	20.7 mega pixel

^a^Not applicable.

### RQ 1: What Types of Hand Dysfunctions Are Studied, and What Clinical Hand Assessment Tools Are Used?

#### Overview

The hand dysfunctions discussed in the 46 articles were classified as an abnormal hand range of motion (ROM; n=18, 39%), hand tremor (n=15, 33%), hand bradykinesia (n=9, 20%), fine hand use decline (n=9, 20%), hypokinesia (n=4, 9%), and hand arthritis–related hand dysfunction (n=2, 4%). A total of 27 (59%) studies used clinical hand assessment tools (Table 3).

**Table 3:** Hand dysfunction type.

Hand dysfunction	Reference
Abnormal range of motion	[24-27,32,35,37-41,45-47,49,59,60,65]
Tremor	[6,9,10,28,31,33,36,42,48,51,52,54,56,61,63]
Bradykinesia	[6,9,11,33,36,43,48,53,54]
Decline of fine motor skills	[9,39,44,51,55,61-64]
Hypokinesia	[30,32,34,58]
Hand arthritis–related hand dysfunction	[29,57]

#### Abnormal Hand ROM

ROM describes how far a joint or muscle can move [67]. The measurement of ROM can indicate joint impairments in patients or the efficacy of rehabilitation programs [67]. Of the 46 studies, 19 (41%) focused on abnormal ROM, 11 (24%) focused on wrist ROM, and 10 (21%) focused on finger ROM. Smartphones were generally placed on the flexor carpi radialis and extensor pollicis longus [25,37,59] to measure wrist ROM and on the distal interphalangeal joint and proximal interphalangeal joint to measure finger ROM [24,25,35,37-39,45,49,60]. In addition, 6 related problems, namely hand injury [24,25,37,38,40,46,65], wrist injury [26,27,46,47,59], stroke [32,37,39,45], after hand surgery [41,60], flexor tendon injury [35], and nerve injury [49], were studied. Most studies (13/19, 68%) showed that the smartphone-based measurement method had the same reliability as the conventional goniometer when evaluating the ROM of healthy people and patients.

#### Hand Tremor

Hand tremor is a rhythmic, involuntary, and oscillatory (ie, rotating around a central plane) movement involving hand distal joints (eg, fingers and wrist) that is regularly recurrent [68]. All studies, except for 1 study on multiple sclerosis (MS), focused on PD hand tremors. For PD hand tremor assessment, the acceleration, rotational velocity, signal shake number and intensity were collected during daily life activities [6,10,28,36,42,51,56,61]. The number of taps or accuracy of each tap was measured during the finger-tapping activity of the smartphone app [33,48,50,52,63]. Smartphone-based hand dysfunction assessment shows satisfactory repeatability and validity when measured against the Movement Disorder Society of Unified Parkinson’s Disease Rating Scale (MDS-UPDRS) [28,33,36,50,52].

#### Hand Bradykinesia

Hand bradykinesia is characterized by slowness, reduced amplitude of movement, and sequence effect [69]. Hand bradykinesia is observed in patients with PD and patients with MS. PD and MS bradykinesia were detected in touch gestures, including finger tapping [9,11,36,43,53,54] and flick and pinch tactile behaviors [48]. The number of tapping trials and finger positions were examined to assess bradykinesia in hands. Daily activities and finger-to-nose tests were performed when holding the smartphone [6,33]. It was found that smartphones were comparable to conventional methods (such as MDS-UPDRS and Modified Bradykinesia Rating Scale) for assessing hand bradykinesia and may be useful in clinical practice [11,33,36,53].

#### Fine Hand Use Decline

Fine hand use refers to the use of small hand muscles to create movements, such as the use of a pencil to draw [70]. A total of 4 diseases were mentioned: PD [9,44,51,55,61,62], stroke [39], MS [63], and Huntington disease [64]. This type of hand dysfunction was assessed through smartphone screen interaction, such as playing games and typing activities [39]. Users’ hold time, flight time, and pressure sequences during smartphone keystroke typing activity were used to quantify fine motor functions [9,44,51,55,62-64]. Studies show that smartphone has the potential to detect PD symptoms from the users’ typing activity, which facilitates the development of digital tools for remote pathological symptom screening [39,44,61].

#### Hypokinesia

Hypokinesia is a decline in muscle strength that causes the muscle to not contract or move as it used to [71]. Three diseases related to this type of hand dysfunction are stroke [32,58], carpal tunnel syndrome (CTS) [30], and hand arthritis [34]. Patients who had a stroke were asked to perform gestures of grasping and floating [32,58] with a sensor glove worn. Hand information, such as finger position and velocity, were collected from patients with CTS as they played a game [30]. Patients with arthritis participated in power, pinch, and tripod grip tasks to capture grip measures [34]. These new methods show high sensitivity and specificity for disease detection and self-assessment [30,34].

#### Hand Arthritis–Related Hand Dysfunction

Arthritis is a common condition and is the most frequent cause of disability in American adults [57]. The most common form of arthritis is osteoarthritis, followed by inflammatory arthritis [72]. A method of analyzing hand dysfunction related to hand arthritis involved capturing photographs of each patient’s hands. The results indicated that this approach could assist in the primary care, clinical assessment, and management of patients with hand arthritis [29].

Hand assessment tools used in the reviewed studies included clinical scales and instruments (Table 4). Clinical hand assessment tools were used for 2 purposes in 32 (70%) of the 46 studies: task design (n=7, 15% studies) and smartphone assessment outcome validation (n=25, 54% studies). The rest of the studies (14/46, 30%) did not mention the clinical tools. MDS-UPDRS was the most used clinical scale (15/46, 33%), while a conventional goniometer was the most used instrument (10/46, 22%) [9,24,35,38,40,41,47,49,59,65]. Some studies used the MDS-UPDRS and the alternative finger-tapping test as reference tasks to set up experiment tasks. The effectiveness and reliability of smartphone-based assessment methods were validated by comparing the results with those of the MDS-UPDRS and manual goniometry.

**Table 4:** Clinical hand assessment tools used.

Clinical scale or instrument			References
**For task design**			
	MDS-UPDRS^a^	[9-11]	
	CAPSIT-PD^b^	[43]	
	AFT^c^	[9,50,54,62]	
	TTT^d^	[9]	
**For outcome validation**			
	MDS-UPDRS	[28,31,33,36,38,39,43,50,52,53,62,64]	
	PDDS^e^	[63]	
	Neuro-QoL^f^	[63]	
	UHDRS^g^	[64]	
	Disease Activity Score-28	[34]	
	PDQ-8^h^	[36]	
	MBRS^i^	[11]	
	Tang criteria	[35]	
	Conventional goniometer	[9,24,35,38,40,41,47,49,59,65]	
	Mechanical tappers	[43]	
	Accelerometer	[61]	
	Electronic digital caliper	[57]	

^a^MDS-UPDRS: Movement Disorder Society of Unified Parkinson’s Disease Rating Scale.

^b^CAPSIT-PD: Core Assessment Program for Surgical Interventional Therapies in Parkinson’s Disease.

^c^AFT: alternating finger tapping.

^d^TTT: time-tapping test.

^e^PDDS: patient-determined disease step.

^f^Neuro-QoL: quality of life in neurological disorders.

^g^UHDRS: Unified Huntington Disease Rating Scale.

^h^PDQ-8: 8-question Parkinson’s Disease Questionnaire.

^i^MBRS: Modified Bradykinesia Rating Scale.

### RQ 2: How Are Smartphone-Based Hand Assessment Tools Applied in Clinical Practice?

Smartphone-based hand assessment has been applied in 4 different ways. It has been used for the measurement of function parameters (ie, wrist and finger ROM and hand strength), the early detection of disease-related dysfunction, real-time assessment during rehabilitation, and function assessment and rating (Table 5).

**Table 5:** Functions of smartphone-based hand assessment tools.

Application setting and task scenario		References
**Measurement**		
	Finger or wrist extension or flexion	[24-27,35,37-41,47,49,59,60]
	Finger implement squeeze and finger forward flexor tendon gliding	[25]
	A grip force–tracking task	[34,45]
	TTT^a^	[9,43]
	RAM^b^, tremor tracker, and CIT^c^	[9]
	Wrist pronation and supination	[65]
**(Early) detection**		
	Daily activity	[42]
	Extended and rest versions of MDS-UPDRS^d^	[10,51]
	Finger-tapping test	[44,51,52,54,55,62]
	Daily motor active tests	[6]
	Flick, drag, pinch, and handwriting gestures	[48]
	Play a game	[30]
	Finger-to-nose test, pronation supination test, and arm-circle exercise	[28]
	Photographic capture of the patient’s hands	[29,57]
	Reaction time test	[52]
**Real-time assessment during rehabilitation**		
	Finger and wrist extension	[26,37,39]
	Wrist flexion, wrist extension, finger implement squeeze, and finger forward flexor tendon gliding	[25]
	A grip force–tracking task	[45]
	Play a game	[39,46]
	Grasping, pinching, and waving	[32]
	Hand grip and flat	[58]
**Function-level rating**		
	Hanging gestures	[28,31,33]
	Finger-to-nose test	[33,61,63]
	Photographic capture of the patient’s hands	[57]
	Grip force–tracking task	[30]
	Extended and rest versions of MDS-UPDRS	[29,50,61]
	Finger-tapping test	[11,33,36,53,63,64]
	Hold the phone	[61]

^a^TTT: time-tapping test.

^b^RAM: rapid alternating movement.

^c^CIT: Cognitive Interference Test.

^d^MDS-UPDRS: Movement Disorder Society of Unified Parkinson’s Disease Rating Scale.

Of the 46 studies, 18 (39%) focused on the measurement of hand function parameters such as wrist ROM [26,27,37,40,41,47,59,65], finger ROM [24,25,35,37-39,45,49], hand gesture [49], hand dexterity [9], or hand grip strength [34]. Hand grip strength measurement and hand dexterity measurement were conducted on smartphones and shown to have good constancy with traditional measurement tools [16,23,38].

Furthermore, 15 (33%) out of the 46 papers focused on dysfunction assessment for early disease detection. Dysfunctions, such as hand tremor (10/46, 22%), hand bradykinesia (3/46, 7%), fine hand use decline (5/46, 11%), and hypokinesia (2/46, 4%), were used as biomarkers for certain diseases, such as PD [6,10,29,30,42,44,48,51,52,54,55,57,61,62], CTS [30], and hand arthritis [57,65]. The detection exhibited high sensitivity and specificity, supporting personalized treatment plan adjustments and enabling early disease diagnosis and optimized management [55].

Among the 46 studies, 14 (30%) concentrated on rating hand dysfunction severity, mostly in PD- or MS-induced hand tremor (8/46, 17%) and bradykinesia (4/46, 9%). The findings demonstrate that smartphones can determine the degree to which the patient is affected by the disease, rating the severity of both the disease and hand dysfunction [45,67,68].

Furthermore, 8 (17%) of the 46 studies explored how smartphones were used for real-time hand function assessment during hand rehabilitation [25,26,32,37,39,45,46,58]. Smartphones provide an interactive interface with guided exercises, therapeutic games, and performance feedback [26,45]. The results of real-time assessment during rehabilitation can help increase patients’ motivation and interest, reduce discontinuity in the rehabilitation process, and lower treatment costs [25,26,32,37,39,45,46,58].

### RQ 3: How Are Smartphones Used to Assess Hand Function?

The literature showed that smartphones had been used in 4 ways for hand function assessment: data collection (38/46, 83% studies), data display (17/46, 37% studies), data transmission (15/46, 33% studies), and data processing (6/46, 13% studies).

#### Data Collection

Data were mainly collected via embedded smartphone sensors or smartphone apps [42]. Accelerometers (12/46, 26%) [15,24,26,28,33,36,42,51,54,56,61,64] were the most used built-in smartphone sensors, followed by smartphone cameras (11/46, 24%) [11,27,29,31,35,40,41,49,53,57,60], gyroscopes (5/46, 11%) [6,10,51,59,64], and goniometers (2/46, 4%) [38,47] (Table 6). Some of the smartphone apps (16/46, 35%) [30,33,43,50,52,63,64] were developed to work as a digital tapper to collect the number of trials and position of each tap during the time-tapping test, and AFT task was used to detect hand use, hand tremor, bradykinesia, or ROM. Accelerometers can collect rich information, including angles and the rotational velocity vector of the finger [24,26]. The sampling rate range of accelerometers was 20 to 100 Hz. By using a smartphone’s camera, the patient’s hand picture can be captured to extract information such as wrist and finger extension and flexion, allowing the measurement of joint ROM or extension [35,41,60]. The camera resolution range was 1920×1080 pixels to 2400×1080 pixels.

**Table 6:** Built-in sensors involving data collection.

Sensor and measurement		App name	References
**Accelerometers**			
	All angles of DIPj^a^, PIPj^b^, and MPj^c^, including the right and left, active and passive, and extensor and flexor positions	Google LLC and EHMROM	[24]
	Still acceleration	HTrembAPP^d^	[42]
	The acceleration vector and the rotational velocity vector	DNM^e^	[15]
	Accelerometer signal	Roche PD Mobile Application (version; Roche), PD Dr, Apkinson, GEORGE, mPower, and mobile accelerometer software	[28,33,36,51,54,56,61,64]
	Orientation, velocity, and motion	HandRehab app	[26]
**Smartphone app**			
	Number, time, velocity, position, consistency, amplitude, and accuracy of each tap	SmT^f^, DNM, mPower, Apkinson, elevateMS, ReHand, GEORGE, and HLTapper	[30,33,36,43,50,52,54,55,61-64]
	150 test parameters	DNM	[9]
	Kinetic tremor and dysmetria in movement	elevateMS	[63]
	Pronation, supination, flexion, and extension	DNM and Angulus app	[40,65]
**Camera**			
	Movement and tremor	Did not use an app	[27,31]
	Hand video	Did not use an app	[11,31,53]
	Hand picture	DNM	[29,35,40,41,49,57,60]
	Joints’ angles and key point’s distance	Did not use an app	[49]
	Extension or flexion of the joint	Did not use an app	[35,41,60]
	Movement of finger	Did not use an app	[60]
	Tapping frequency, amplitude, speed, or rhythm	Did not use an app	[11,53]
**Gyroscope**			
	Gyroscope data in discrete time	DNM	[10]
	Gyroscope signal	Roche PD Mobile Application (version 1; Roche) and GEORGE	[51,64,66]
	Height, rotation, slope, and acceleration	Gyroscope	[59]
**Goniometer**			
	Finger flexion at MCPj, PIPj, and DIPj and flexion angles of the finger	Goniometer	[38]
	Wrist flexion, extension, supination, and pronation ROM^g^	Compass app	[47]
**GPS**			
	Orientation, velocity, and motion	HandRehab app and newly created smartphone apps	[26]
**Microphone**			
	Voice	Roche PD Mobile Application (version 1)	[6]
**Pressure sensor**			
	Pressure-based features	Custom Android app (the name of the app was not mentioned)	[48]
	Finger pressure	DNM	[46]
**IMU^h^**			
	IMU–based features	Custom Android app (the name of the app was not mentioned)	[48]

^a^DIPj: distal interphalangeal joint.

^b^PIPj: proximal interphalangeal joint.

^c^MPj: metacarpophalangeal joint.

^d^HTrembAPP: Hand Trembling Detector App.

^e^DNM: did not mention.

^f^SmT: smartphone tapper.

^g^ROM: range of motion.

^h^IMU: inertial measurement unit.

#### Data Display

Data display (17/46, 37%) included the display of raw data (12/46, 26%) [24,26,28,32,34,37,42,45,51,55,58,61], visual instructions (10/46, 22%) [25,26,28,30,37,39,46,55,63,64], and information notification [10,61] (2/46, 4%). Data were frequently displayed in the text form [28,32,34,42,45,51,55,61] and graphic form [24,26,37,58]. Test details, such as date and patient information [26,42,45], were usually displayed. Assessment feedback was also displayed in the form of results or scores [25,45]. The real-time feedback displayed included hand motion data [28,45], virtual 3D representation of finger posture [26], and interactive game interfaces [39].

#### Data Transmission

Data transmission describes how data are transmitted between smartphones and external devices (Table 7). Due to limited data processing capacity, smartphones generally send data to other resources through Bluetooth, USB dongles, and Wi-Fi for data processing and storage [6,39,43]. Of the 46 studies, 12 (26%) transmitted the data to a cloud server through a unidirectional transfer, meaning data only flowed in 1 direction. Among these 12 studies, 7 (58%) developed a smartphone app to receive the built-in sensor data [6,10,26,28,32,43,61], and the other 5 (42%) designed a smartphone app to receive the training data from external devices (ie, gloves) [25,39,46,58,62]. A total of 3 (%) of the 46 papers reported that smartphones transmitted data with an external device via bidirectional communication [32,45,58], indicating smartphones can send and receive data in both directions. Furthermore, 2 (%) of the 46 papers discussed data privacy and security and referred to Health Insurance Portability and Accountability Act regulations [32].

**Table 7:** The objects involved in data transmission.

Receiver		References
**Remote server**		
	Computer	[10,56]
	Google Drive	[43]
	Cloud storage facility	[6]
	Cloud computing	[28,47]
	Remote server	[32,61,62]
	Physician	[6,25,26,46]
**External device**		
	Glove	[32,39,46,58]
	HandMATE device	[39,45]

#### Data Processing

Data processing involves the use of smartphones as terminals to analyze, manipulate, and transform raw data into useful information or machine-readable content [39]. Among the 46 studies, 6 (13%) used a smartphone app to process data [24-26,32,39,42], and 1 (2%) reported the smartphone’s processing power [24]. The smartphone processed motion data collected from built-in sensors and external devices. Data collected from built-in sensors, such as ulnar and radius deviations, were converted into ROM and total active motion [24,39,42]. Data from external devices’ sensors, such as flex-sensor signals and electromyography, were transformed into flexion and extension angles (in degrees) [26,32]. One of the studies extracted the features from electromyography sensors and then fed them to an ML algorithm for further gesture recognition on smartphone apps [25].

#### Use of Smartphones for Multiple Functions

A total of 21 (46%) of the 46 studies designed smartphones integrating more than one of the functions mentioned earlier. The most frequent combination was using a smartphone for data transmission and data display [25,26,28,32,39,45,46,58,61] (Table 8). A total of 8 (17%) studies combined ≥3 functions [24-26,28,32,39,42,61]. For example, in the study by Bercht et al [25], the smartphone was designed to integrate processing capabilities, enabling the real-time reception of game information from the glove’s flex sensor and then display of the information on the smartphone screen after local data processing.

**Table 8:** Use of smartphones for multiple purposes.

Study, year	Data collection	Data processing	Data transmission	Data display
Matera et al [26], 2016	✓	✓	✓	✓
Miyake et al [24], 2020	✓	✓		✓
García-Magariño et al [42], 2016	✓	✓		✓
Bercht et al [25], 2012		✓	✓	✓
Janarthanan et al [39], 2020		✓	✓	✓
Pan et al [28], 2015	✓		✓	✓
Orozco-Arroyave et al [61], 2020	✓		✓	✓
Sarwat et al, 2021 [32]		✓	✓	✓
Kostikis et al [10], 2015	✓		✓	
Lee et al [43], 2016	✓		✓	
Lipsmeier et al [6], 2018	✓		✓	
Sandison et al [45], 2020			✓	✓
Halic et al [46], 2014			✓	✓
Koyama et al [30], 2021	✓			✓
Chén et al [51], 2020	✓			✓
Arroyo-Gallego et al [62], 2017	✓			✓
Pratap et al [63], 2020	✓		✓	
Waddell et al [64], 2021	✓			✓
Mousavi et al [56], 2020	✓			✓
Lee et al [55], 2016	✓		✓	
Hidayat et al [58], 2015	✓			✓

### RQ 4: What Statistics or ML Algorithms Are Used for Hand Function Assessment?

#### Overview

Among the 46 studies, 39 (85%) used statistical methods to process the hand motion data, including parameters such as tapping speed, error, and speed during smartphone screen interaction; 20 (43%) applied ML to analyze the raw data or statistical features; and 17 (37%) used both statistical and ML methods. By contrast, 4 (9%) studies used neither statistics nor ML for data analysis [37,39,47,59].

#### Statistical Methods

Overall, 21 types of statistical methods were used to process 6 types of hand motion raw data (Table 9). The most used method was summary statistics (23/46, 50%), followed by normalization (7/46, 15%) and Fourier transform (6/46, 13%).

**Table 9:** Studies classified by statistical methods.

Data processed and statistical method		References
**Data collected during the smartphone screen interaction (ie, tapping speed, error, speed, path, pressure, and distance)**		
	Pythagorean theorem	[43]
	Normalization	[33,48,54,61,62,64]
	Bootstrap multiple regression	[9]
	Summary statistics (range, mean, median, and SD)	[11,30,36,43,50,52,53,55,62]
	Akaike information criterion	[9]
	Fourier transform	[11,33,53]
**Accelerometer values and rotational velocity vector**		
	ObtainDirection	[42]
	ObtainAlpha	[42]
	Band-pass filter	[10,64]
	Spectral analysis	[10]
	Fourier transform	[10,28]
	Summary statistics (range, mean, median, and SD)	[34]
	Mass univariate	[51]
	Feature-wise correlation test	[51]
	Regularization	[51]
	Butterworth high-pass filter	[33]
	EMD^a^	[56]
**Smartphone video or picture**		
	Fourier transform	[31]
	Normalization	[57]
	Summary statistics (minimum, maximum, mean, median, and SD)	[60]
	One-hot encoding categorical and scaling numerical responses	[29]
	Savitzky-Golay filter	[11]
**Initiating, terminating flexion, extension, and ROM^b^**		
	RMS^c^ error	[27,32,35,40,45]
**FSR^d^, IMU^e^, or pressure sensor signals**		
	Ōtsu’s 11 binarization	[41]
	RMS error	[45]
	Summary statistics (range, mean, median, and SD)	[32,37]
	SMA^f^ filtering	[58]
**Variables for model prediction (ie, age, sex, and occupation)**		
	Linear mixed models	[59]
	Multiple linear regression	[9]

^a^EMD: empirical mode decomposition.

^b^ROM: range of motion.

^c^RMS: root mean square.

^d^FSR: force sensing resistor.

^e^IMU: inertial measurement unit.

^f^SMA: simple moving average.

#### ML Methods

In total, 16 types of ML methods were identified (Table 10). They were applied for 4 purposes: disease detection, disease severity evaluation, disease prediction, and feature aggregation. Support vector machines (SVMs) were the most used ML method [10,28,48,49,53,56,62]. The input features of SVMs were preprocessed acceleration signals, such as the sums of squared magnitudes [10] and path- or time-based features [48]. Tian et al [60] reported SVMs as a reliable ML method for early PD detection and multivariate classification with 0.89 sensitivity and 0.88 specificity. Gu et al [49] reported the highest gesture classification accuracy of 1, with a sensitivity of 1 and specificity of 1.

Among the 46 studies, 5 (11%) applied logistic regression for disease severity classification and prediction and hand gesture discrimination [32,51,53,54,62]. The spatiotemporal features from the pixel coordinate data during finger tapping and accelerometer waveforms were the input for this ML method. Logistic regression showed an average accuracy of 88.5% (SD 8.03%; grasp), 83% (SD 10.9%; pinch), and 86.5% (SD 12.57%; wave) [32] and an accuracy of 0.61 and area under the curve (AUC) of 0.59 in PD prediction [53].

Of the 46 studies, 3 (7%) [29,44,54] exploited convolutional neural networks to distinguish patients with PD from healthy controls based on hold time, flight time, and pressure sequences [44]. Convolutional neural networks exploited the finger-tapping rate data for PD severity identification with an AUC of 0.64 and accuracy of 0.62 [54]. They also worked as the base layer for training 2 image preprocessing models and for discriminating PD tremors from other types of tremors with 95% agreement with the accelerometer [29].

Among the 46 studies, 7 (15%) [10,32,48,49,51,53,62] compared the classification performance of different ML algorithms. For example, Kostikis et al [10] applied decision tree (DT), Naive Bayes, C4.5 DT, and a bagged ensemble of DTs for distinguishing patients with PD from healthy participants based on PD hand tremor features. Bagged ensemble of DTs performed better than other classifiers, with an accuracy of 0.90 for the healthy group and 0.82 for the PD group and an AUC of 0.94.

**Table 10:** Studies classified by MLa algorithms.

ML and feature			Validity and accuracy		References
**SVM^b^**					
	Magα^c^, magω^d^, sdα^e^, and mAmpω^f^	Distinguishing patients with PD^g^ from healthy participants: sensitivity=0.56 and specificity=1		[10]	
	Path-based, time-based, pressure-based, and IMU^h^-based features and additional features for handwriting gestures and pinch gestures	In healthy controls: sensitivity=0.89 and specificity=0.88		[48]	
	The total, peak and fraction power and average acceleration of the motion data	PD hand resting tremor detection: sensitivity=0.77 and accuracy=0.82		[28]	
	Angles of the MCPj^i^, PIPj^j^, DIPj^k^, and CMCj^l^ of fingers; webspace; etc	Highest gesture classification: accuracy=1, sensitivity=1, and specificity=1		[49]	
	SFS^m^ to select the best feature from the mean, SD, skewness, etc, from accelerometer signals	Tremor activity identified with the highest accuracy of 0.91, specificity of 0.90, and sensitivity of 0.90		[56]	
	Touchscreen typing features: covariance, skewness, and kurtosis analysis of the timing information	The typing feature aggregated with an AUC^n^ of 0.88 (linear-SVM)		[62]	
	Tapping frequency, amplitude, energy spectral density, and peak-to-peak variability	PD diagnosis predicted with an accuracy of 0.63 and AUC of 0.60 (linear-SVM)		[53]	
	Tapping frequency, amplitude, energy spectral density, and peak-to-peak variability	PD diagnosis predicted with an accuracy of 0.69 and AUC of 0.68 (SVM-RBF^o^)		[53]	
**Logistic regression**					
	The mean, RMS^p^, SMA^q^, and SD for each axis of the accelerometer and gyroscope	Patient performance assessed with average accuracy of 88.5% (SD 8.03%; grasp), 83% (SD 10.9%; pinch), and 86.5% (SD 12.57%; wave)		[32]	
	Touchscreen typing features: covariance, skewness, and kurtosis analysis of the timing information	The typing feature aggregated with an AUC of 0.87		[62]	
	Tapping frequency, amplitude, energy spectral density, and peak-to-peak variability	PD diagnosis predicted with an accuracy of 0.61 and AUC of 0.59		[53]	
	13 spatiotemporal features from the pixel coordinate data about speed, rhythm, accuracy, and fatigue and 28 features from 3 accelerometer waveforms, frequency, and temporal domains	PD severity classified with an AUC of 63.1 (SD 2.11) accuracy of 59.5 (SD 0.96)		[54]	
	Features selected according to formulas and parameters	Patients with PD distinguished from healthy participants with an accuracy of 0.94, sensitivity of 0.95, and specificity of 0.94 (multivariate logistic regression)		[51]	
**CNN^r^**					
	4 statistical features from HT^s^, FT^t^, and pressure sequences	Classification of patients with PD and healthy controls: in the clinic, mean performance=0.89, sensitivity=0.79, and specificity=0.79; in the wild, mean performance=0.79, sensitivity=0.74, and specificity=0.78		[44]	
	12 features, such as sex, age, and the duration of symptom	Discriminant PD tremor with 95% agreement with accelerometer		[29]	
	Raw data of finger tapping	PD severity identified with an AUC of 63.5 (SD 1.56) and accuracy of 62.1 (SD 0.95)		[54]	
**RF^u^**					
	Angle of fingers’ MCPj, PIPj, DIPj, and CMCj; webspace; etc	Highest gesture classification: accuracy=1, sensitivity=1, and specificity=1		[49]	
	Mean, SD, and median acceleration	In discriminating participants with PD from controls, sensitivity=0.96 (SD 0.2) and specificity=0.97		[52]	
	13 spatiotemporal features from the pixel coordinate data about speed, rhythm, accuracy, and fatigue and 28 features from 3 accelerometer waveforms, frequency, and temporal domains	PD severity identified with an AUC of 64.1 (SD 1.08) and accuracy of 60.2 (SD 1.56)		[54]	
**Linear regression**					
	Magα, magω, sdα, and mAmpω	Patients with PD distinguished from healthy participants with a sensitivity of 0.74 and specificity of 1		[10]	
	Angle of fingers’ MCPj, PIPj, DIPj, and CMCj; webspace; etc	Highest gesture classification: accuracy=1, sensitivity=1, and specificity=1		[49]	
**AdaBoost**					
	Magα, magω, sdα, and mAmpω	Patients with PD distinguished from healthy participants with a sensitivity of 0.83 and specificity of 0.85		[34]	
	Touchscreen typing features: covariance, skewness, and kurtosis analysis of the data timing information	The typing feature aggregated with an AUC of 0.82		[62]	
**KNN^v^**					
	Time domain: the signal length, mean value, RMS value, number of vertices, and number of baseline crosses; frequency domain: fundamental frequency, region length, and Fourier variance	Validated with self-defined hand gesture performance classification standards with an accuracy of >95%		[25]	
**NB^w^**					
	Magα, magω, sdα, and mAmpω	Patients with PD distinguished from healthy participants with a sensitivity of 0.56% and specificity of 1		[10]	
	Tapping frequency, amplitude, energy spectral density, and peak-to-peak variability	PD diagnosis predicted with an accuracy of 0.69 and AUC of 0.70		[53]	
**XGBoost^x^**					
	Features selected according to formulas and parameters	Patients with PD distinguished from healthy participants with an accuracy of 0.81, a sensitivity of 0.83, and a specificity of 0.9		[51]	
	The mean, RMS, SMA, and SD for each axis of the accelerometer and gyroscope	Patient performance assessed with average accuracy of 88% (SD 9.88%; grasp), 83.5% (SD 7.74%; pinch), and 82% (SD 14.71%; wave)		[32]	
**C4.5 DT^y^**					
	Magα, magω, sdα, and mAmpω	Patients with PD distinguished from healthy participants with a sensitivity of 0.83 and specificity of 0.75		[10]	
**BagDT^z^**					
	Magα, magω, sdα, and mAmpω	Patients with PD distinguished from healthy participants with a sensitivity of 0.82 and specificity of 0.90		[10]	
**DT**					
	Magα, magω, sdα, and mAmpω	Patients with PD (accuracy rate 82%) distinguished from healthy people (accuracy rate 90%)		[10]	
**HAR^aa^**					
	Sustained phonation: MFCC2^ab^; rest tremor: skewness; postural tremor: total power; finger tapping; balance: mean velocity; and gait: turn speed	Unlabeled PD activity test data: PD balance activity test: 99.5%; gait activity test: 96.9%; and distinguishing between resting and gait activities: 98%		[6]	
**Anomaly detection and an autoencoder**					
	The position, time, or velocity of the thumb movement	Participants with and participants without CTS^ac^ classified with a sensitivity of 0.94, a specificity of 0.67, and an AUC of 0.86		[30]	
**Elastic net**					
	Features selected according to formulas and parameters	Patients with PD distinguished from healthy participants with an accuracy of 1, a sensitivity of 0.95, and a specificity of 1		[51]	
**DNN^ad^**					
	13 spatiotemporal features from the pixel coordinate data, including speed, rhythm, accuracy, and fatigue, and 28 features from 3 accelerometer waveforms, frequency, and temporal domains	PD severity classified with an AUC of 65.7 (SD 1.05) and accuracy of 61.2 (SD 1.07)		[54]	

^a^ML: machine learning.

^b^SVM: support vector machine.

^c^magα: the sums of squared magnitudes of the acceleration.

^d^magω: the sums of squared magnitudes of the rotation rate vector.

^e^sdα: the sums of absolute differences in the acceleration vector.

^f^mAmpω: the maximum sums of the 3 axial components of the rotation vector ω calculated by Fourier transform.

^g^PD: Parkinson disease.

^h^IMU: inertial measurement unit.

^i^MCPj: metacarpophalangeal joint.

^j^PIPj: proximal interphalangeal joint.

^k^DIPj: distal interphalangeal joint.

^l^CMCj: carpometacarpal joint.

^m^SFS: feature selection algorithm.

^n^AUC: area under the curve.

^o^RBF: radial basis function.

^p^RMS: root mean square.

^q^SMA: simple moving average.

^r^CNN: convolutional neural network.

^s^HT: hold time.

^t^FT: flight time.

^u^RF: random forest.

^v^KNN: K-nearest neighbor.

^w^NB: naive Bayes.

^x^XGBoost: extreme gradient boosting.

^y^DT: decision tree.

^z^BagDT: bagged ensemble of decision trees.

^aa^HAR: human activity recognition.

^ab^MFCC2: mel-frequency cepstral coefficient2.

^ac^CTS: carpal tunnel syndrome.

^ad^DNN: deep neural network.

## Discussion

To the best of our knowledge, this is the first systematic review on the primary design ideas and development of smartphone-based technologies for hand function assessment.

### RQ 1: What Types of Hand Dysfunctions Are Studied, and What Assessment Inventory Tools Are Used?

In the literature, smartphones only assessed 6 types of hand dysfunctions, namely abnormal ROM, tremor, bradykinesia, fine motor skill decline, hypokinesia, and hand arthritis–related hand dysfunction. The reason might be that smartphones are limited in capturing the complexity and variety of hand movements to measure all aspects of clinically relevant hand functions [73]. Other types of hand dysfunctions such as decreased grip strength, altered sensation, and impaired coordination are important biomarkers clinically, requiring the future development of smartphones to collect related parameters [74].

ROM is a critical and objective measurement that can reflect various diseases, such as arthritis, trauma, and stroke [75]. Abnormal ROM was the most studied smartphone-based hand function assessment [24-27,32,37-41,45-47,49,51,59,60,65], indicating the advantages of smartphones in obtaining ROM parameters. Therefore, the further development of smartphones to achieve better accuracy and reliability in capturing ROM is warranted. With the advancement of built-in accelerometers and gyroscopes in smartphones, capturing and analyzing hand ROM data have become more accessible [75,76]. Furthermore, smartphones can accurately measure both dynamic ROM and static ROM, providing good potential for long-term monitoring even without the presence of professionals [27].

PD is the most studied disease that causes hand dysfunction. PD can cause multiple hand dysfunctions, such as tremors [6,9,10,42,48], bradykinesia [6,9,43,48], abnormal ROM [37,39,45], and fine hand use decline [44]. It provides evidence that smartphones have the potential to provide a comprehensive assessment platform for multiple hand dysfunctions [9,42-44].

In addition, chronic neurodegenerative diseases, such as PD, exhibit progressive symptoms that require continuous monitoring [7]. However, existing clinical assessment tools, such as MDS-UPDRS, tend to be subjective, time constrained, and time consuming [77]. Smartphone apps could exploit the multiple built-in sensors in smartphones to detect changes indicative of the disease progression or treatment response [78-82], indicating that smartphones can be prosperous tools for managing chronic hand dysfunction in the long run.

Above all, for a reliable clinical application of hand dysfunction assessment, the following should be achieved:

1. Gold standards should be established and validated, specific to the smartphone as an assessment platform.
2. Smartphone assessment should be customizable according to an individual’s condition and rehabilitation expectations [83].
3. Smartphone assessment procedures and tasks should adhere to the operational specifications of the clinical assessment criteria [2,84].
4. An individualized rehabilitation plan should be generated from the assessment and evaluated in real-time pace to monitor the individual’s rehabilitation progress.

### RQ 2: How Are Smartphone-Based Hand Assessment Tools Applied in Clinical Practice?

Real-time assessment during hand rehabilitation is beneficial in clinical practice because it allows the modification of the rehabilitation tasks and goals according to an individual’s specific needs and ongoing recovery progress [85]. In our review, studies on real-time smartphone-based assessment were primarily conducted between 2016 and 2022, indicating an emerging trend focusing on real-time hand assessment. A potential technical challenge may lie in identifying the best sensor configuration and feature extraction method for hand function assessment [6,84].

The early detection of a degenerative disease through hand assessment is important because it can help slow down further disease progression [86]. The reviewed literature discussed conditions such as PD and CTS [34,36,37]. Future work could use smartphones for biomarker acquisition to monitor disease-relevant physiological and behavioral symptoms and provide personalized rehabilitation guidance [87-89]. The use of smartphones for biomarker acquisition offers advantages, including portability, accessibility, affordability, noninvasiveness, and continuous monitoring, benefiting both patients and clinicians [90]. However, challenges exist in terms of data quality, reliability, and privacy concerns [91].

### RQ 3: How Are Smartphones Used to Assess Hand Function?

Smartphones were mostly used for data collection. With more sensors embedded in smartphones, richer and more dimensional data can be collected for function measurement. For example, the resolution of smartphones’ built-in camera is between 1920×1080 and 2400×1080 pixels, which is higher than the commonly used camera resolution in clinical settings, which typically ranges from 1280×720 to 1920×1080 pixels [8]. Compared to smartwatches and ring-shaped sensors, smartphones are more indispensable in people’s daily lives, making them an easily available assessment tool and requiring no extra investment like others [92]. While webcams provide high resolution and frame rates, they rely on a stable internet connection and can potentially raise privacy and security concerns [93]. In comparison, smartphones can collect data offline and protect the patient’s privacy by encrypting data, anonymizing personal information and storing data locally [40]. This also shows that smartphones, as general-purpose devices, do not require excessive hardware requirements, are available at a low cost, and are easy to access. Smartwatches and wearables usually feature multiple sensors similar to those found in smartphones, allowing for the collection of hand motion and physiological data with real-time feedback. However, their functionality is confined by a fixed position of the body, resulting in the limited scope of data collection [14]. In contrast, smartphones, being portable devices, are not constrained by fixed positions, granting convenience and flexibility for hand dysfunction assessment. Ring-shaped sensors offer high precision and accuracy and provide real-time data. However, their use may be limited due to comfort and portability constraints [16]. Smartphones are equipped with data processing modules, which can analyze and process data in real time, providing better accuracy at the same cost [94]. In terms of user experience, as a more familiar product, smartphones reduce the users’ learning cost and provide a more convenient, personalized, and friendly hand dysfunction evaluation experience, which helps improve user participation and satisfaction [19]. However, one of the weaknesses of using a smartphone for data collection may be data errors or biases due to the smartphone user’s lack of training, supervision, and quality control [95].

**Figure 2:**
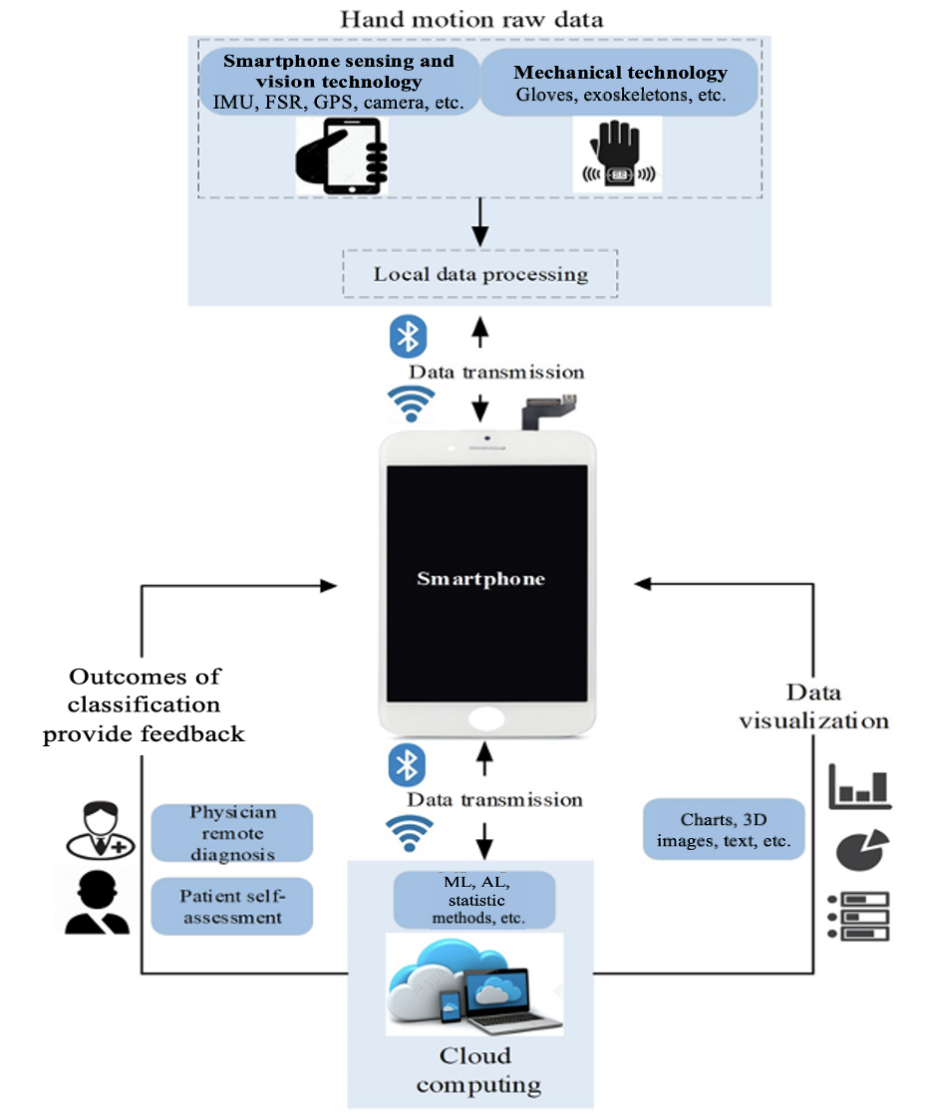
The primary design ideas for the development of smartphone-based hand function assessment technology. AI: artificial intelligence; FSR: force sensing resistor; IMU: inertial measurement unit; ML: machine learning.

Using smartphones for data processing was the least mentioned in the studies [24-26,32,39,42]. The benefits of smartphone data processing are manifold, including mobility, real-time processing, and interactive nature [96]. This empowers users to access and process data at any time, receive real-time feedback, and seamlessly interact with their smartphones, regardless of location [97]. Despite the advantages, there are also obstacles to overcome, including short battery life, limited storage capacity, and weak processing power [98]. Therefore, most of our reviewed studies focused on the wireless transmission of data to computers or the cloud for subsequent data processing [6,10,39,46]. This approach would allow for efficient data management and processing without consuming the limited storage space available in smartphones (Figure 2) [10].

In this review, among the 46 studies, 7 (15%) exclusively involved healthy participants, while 23 (50%) recruited both patients and healthy participants. Consequently, 65% (30/46) of the studies included healthy participants, marking a noteworthy finding. In smartphone-based hand dysfunction assessment, incorporating baseline data from healthy participants is important for several reasons [37-41,59,60]. First, a standard reference range is typically derived from data collected from healthy participants, which could enable a more precise evaluation of a patient’s hand dysfunction. By comparing the hand function of patients to that of healthy participants, potential abnormalities can be identified more effectively, assisting in the accurate diagnosis of issues and facilitating the implementation of appropriate treatments [6,10,11,29,42-55,61-65]. Second, during the rehabilitation process, the patient’s recovery progress and improvement can be quantified by comparing against data from health people [45,46]. The effectiveness of the treatment can be more accurately assessed, and rehabilitation protocols could be adjusted for better outcomes. Third, it’s necessary to establish a normal reference range from healthy participants, including different ages, sex, and demographic characteristics. A broader set of data is available, ensuring that assessments are not limited to a specific group and can cover a broader population, resulting in a complete and more comprehensive understanding of hand function assessment [99]. In summary, remote assessment platforms have been developed for a wide range of users, including professionals, caregivers, and patients [2,10]. However, certain aspects need to be considered when using smartphones for hand assessment. They are as follows [28,100-102]:

1. Establishing standardized data formats is of utmost importance to ensure compatibility and consistency in data analysis. Inconsistent data formats can pose challenges in data analysis, making it difficult to compare and analyze data obtained from various smartphones.
2. It is necessary to ensure the robustness of smartphone processors or network connections. The effectiveness of the smartphone processor and network can impact the frequency of data updates, which may result in delays when acquiring and displaying real-time data.
3. It is necessary to consider privacy and security. It is important to prioritize data security and privacy by implementing app-appropriate encryption measures during data transmission to mitigate potential ethical and legal issues and ensure compliance with relevant data-protection regulations.

### RQ 4: What Statistics or ML Algorithms Are Used for Hand Function Assessment?

Statistical methods (39/46, 85%) were more commonly used than ML methods (20/46, 43%). The most commonly used statistical method was summary statistics such as mean and SD. Summary statistics offer concise insights into data, facilitating comparisons and simplifying analysis [103]. However, they can be subjective, relying on expert experience, and may distort information [104]. In addition, due to the multiple independent variables present in hand function assessment [83], it is important to consider statistical methods that are capable of analyzing a multifactor model, such as multiple linear regression [105].

ML methods have been increasingly used in various health care apps [106]. In the studies in our review, ML methods were mainly used for detecting and classifying patient hand posture, analyzing and classifying behavior patterns (ie, tremor, bradykinesia, and ROM), and identifying disease severity and prediction. Our review found SVMs to be the most commonly used ML algorithm, particularly for disease classification. This may be attributed to the fact that SVMs are capable of effectively addressing multi-dimensional data with small sample sizes while providing a good generalization performance and the ability to work with the primary processing stage data [107]. The main limitation of the SVM algorithm is its inability to handle multiclass classification problems without additional modifications or extensions [108].

### Strengths and Limitations of the Study

The strengths of this review are as follows: (1) the relevant database searches were conducted in a comprehensive and reproducible manner; (2) this was the first review that aimed to comprehensively discuss the role of smartphones and their functionalities in hand assessment from a holistic perspective; and (3) this review provides an analytical demonstration of the technical feasibility and advantages of using smartphones for hand function assessment across various domains, including sensor support, clinical practice, and application scenarios. It recommends potential directions for future studies in this field, such as multisensor fusion, gold-standard establishment, real-time assessment, and ML algorithms for data analysis exploration. This review also has some limitations. First, given that smartphone-based hand function assessment is at its nascent stage, the number of relevant studies is limited. This may contribute to a lack of sufficient evidence, completeness, and comprehensiveness in research materials, posing challenges in supporting viewpoints, drawing conclusions, and gaining a comprehensive understanding of the field. Second, this review encompassed only studies in the English language. Third, due to the exploratory and developmental nature of this topic, the literature quality varied, with potential limitations, such as inconsistency and a lack of high-quality reference studies and as well as meta-analyses.

### Conclusions and Future Research

This systematic review focused on how smartphones are used for hand function assessment. It covered the evaluation and measurement of hand dysfunction caused by various diseases, different embedded smartphone sensors, and statistical and artificial intelligence methods for hand function assessment. The evidence demonstrated that smartphones could facilitate a convenient, inexpensive, and reliable hand-functional assessment [9,10,44]. Future research could (1) explore how to develop a gold standard for smartphone-based hand function assessment; (2) take advantage of smartphones’ integrated systems with multiple sensors to collect patients’ data in various dimensions to assess hand function holistically; and (3) develop ML methods that are more suitable for processing data collected by smartphones. On the basis of the growing capabilities of smartphones for data collection and analysis, digital technology holds promise for bringing revolutionary changes to hand function assessment.

## Appendix

Multimedia Appendix 1 PRISMA (Preferred Reporting Items for Systematic Reviews and Meta-Analyses) checklist.

Multimedia Appendix 2 Search strategy.

Multimedia Appendix 3 Mixed Methods Appraisal Tool matrix.

## Abbreviations

CTS: carpal tunnel syndrome
DT: decision tree
MDS-UPDRS: Movement Disorder Society of Unified Parkinson’s Disease Rating Scale
ML: machine learning
MS: multiple sclerosis
PD: Parkinson disease
PRISMA: Preferred Reporting Items for Systematic Reviews and Meta-Analyses
ROM: range of motion
RQ: research question
SVM: support vector machine

## Data Availability

The data sets generated during and analyzed during this study are available from the corresponding author on reasonable request.
