# Supplementary material for: Smartphone-Based Hand Function Assessment: Systematic Review
Source: J Med Internet Res. 2024 Sep 16;26:e51564. doi: 10.2196/51564 (PMC11443181; doi:10.2196/51564)
Supplement: Multimedia Appendix 2 [file jmir_v26i1e51564_app2.docx]

**Supplement A: Search Strategies
Database: MEDLINE(R) ALL <1946 to June 07, 2023>
Platform: Ovid
Date Searched: 2023-06-08**

<https://uhn.idm.oclc.org/login?url=http://ovidsp.ovid.com/ovidweb.cgi?T=JS&NEWS=N&PAGE=main&SHAREDSEARCHID=2frnT240hCYcz6QVZfoZ3S2snmdkrpQTmI0F4KBhgUz8pbGS6NJRl4byetwEDsgC5>

**Search Strategy:**
**1**  exp Cell Phone/ (22218)
**2**  smartphone/ (9016)
**3**  (smartphone? or smart phone?).tw,kf. (24276)
**4**  (cellphone? or cell phone? or cellular phone?).tw,kf. (4843)
**5**  mobile phone?.tw,kf. (13836)
**6**  Mobile Applications/ (11388)
**7**  (app or apps).tw,kf. (43495)
**8**  ((mobile or portable electronic or portable software) adj application?).tw,kf. (5917)
**9**  mobile health.tw,kf. (8483)
**10**  mhealth.tw,kf. (9158)
**11**  mobile-based.tw,kf. (825)
**12**  phone-based.tw,kf. (1655)
**13**  or/1-12 (89734)
**14**  exp Hand/ (87480)
**15**  exp Hand Strength/ (20272)
**16**  exp Hand Injuries/ (19306)
**17**  ((hand? or handgrip? or finger? or thumb? or forefinger? or pinky or pinkies or metacarpus or metacarpal?) adj6 (dexterity or dexterous* or grip or gripping or grasp or grasping or tap or tapping or strength* or flex* or extent* or extend* or movement* or angle? or pinch)).tw,kf. (43254)
**18**  ((hand? or finger? or thumb? or forefinger? or pinky or pinkies or metacarpus or metacarpal?) adj6 (assess* or function* or therap* or rehab* or analy* or "motor skill?" or measur* or detect*)).tw,kf. (58897)
**19**  14 or 15 or 16 or 17 or 18 (178082)
**20**  13 and 19 (699)
**21**  20 not (exp animals/ not humans.sh.) (686)
**22**  limit 21 to english language (676)

**Database: EBM Reviews - Cochrane Central Register of Controlled Trials <May 2023>
Platform: Ovid
Date Searched: 2023-06-08**
**1**  exp Cell Phone/ (3074)
**2**  smartphone/ (1005)
**3**  (smartphone? or smart phone?).tw,hw. (7700)
**4**  (cellphone? or cell phone? or cellular phone?).tw,hw. (1894)
**5**  mobile phone?.tw,hw. (4011)
**6**  Mobile Applications/ (1544)
**7**  (app or apps).tw,hw. (8376)
**8**  ((mobile or portable electronic or portable software) adj application?).tw,hw. (4066)
**9**  mobile health.tw,hw. (1980)
**10**  mhealth.tw,hw. (2034)
**11**  mobile-based.tw,hw. (368)
**12**  phone-based.tw,hw. (901)
**13**  or/1-12 (20266)
**14**  exp Hand/ (2860)
**15**  exp Hand Strength/ (2073)
**16**  exp Hand Injuries/ (327)
**17**  ((hand? or handgrip? or finger? or thumb? or forefinger? or pinky or pinkies or metacarpus or metacarpal?) adj6 (dexterity or dexterous* or grip or gripping or grasp or grasping or tap or tapping or strength* or flex* or extent* or extend* or movement* or angle? or pinch)).tw,hw. (9535)
**18**  ((hand? or finger? or thumb? or forefinger? or pinky or pinkies or metacarpus or metacarpal?) adj6 (assess* or function* or therap* or rehab* or analy* or "motor skill?" or measur* or detect*)).tw,hw. (11524)
**19**  14 or 15 or 16 or 17 or 18 (19072)
**20**  13 and 19 (286)
**21**  limit 20 to (english language or no language specified) (284)
**22**  limit 21 to trial registry record (178)
**23**  21 not 22 (106)

**Database: Embase Classic+Embase <1947 to 2023 June 07>
Platform: Ovid
Date Searched: 2023-06-08**

**Search Strategy:**
**1**  exp mobile phone/ (47525)
**2**  smartphone/ (26260)
**3**  (smartphone? or smart phone?).tw,kf. (32062)
**4**  (cellphone? or cell phone? or cellular phone?).tw,kf. (6688)
**5**  mobile phone?.tw,kf. (15750)
**6**  exp mobile application/ (24951)
**7**  (app or apps).tw,kf. (60437)
**8**  ((mobile or portable electronic or portable software) adj application?).tw,kf. (7836)
**9**  mobile health.tw,kf. (8593)
**10**  mhealth.tw,kf. (8708)
**11**  mobile-based.tw,kf. (976)
**12**  phone-based.tw,kf. (2194)
**13**  or/1-12 (125218)
**14**  exp hand/ (109968)
**15**  exp hand strength/ (39885)
**16**  exp hand grip/ (12943)
**17**  exp hand injury/ (21422)
**18**  hand disease/ (6154)
**19**  exp hand disease/ (74665)
**20**  ((hand? or handgrip? or finger? or thumb? or forefinger? or pinky or pinkies or metacarpus or metacarpal?) adj6 (dexterity or dexterous* or grip or gripping or grasp or grasping or tap or tapping or strength* or flex* or extent* or extend* or movement* or angle? or pinch)).tw,kf. (61056)
**21**  ((hand? or finger? or thumb? or forefinger? or pinky or pinkies or metacarpus or metacarpal?) adj6 (assess* or function* or therap* or rehab* or analy* or "motor skill?" or measur* or detect*)).tw,kf. (84851)
**22**  or/14-21 (297315)
**23**  13 and 22 (1460)
**24**  23 not ((exp animal/ or nonhuman/) not exp human/) (1400)
**25**  limit 24 to english language (1385)
**26**  limit 25 to medline (285)
**27**  25 not 26 (1100)
**28**  limit 27 to (conference abstract or "conference review") (458)
**29**  27 not 28 (642)

**Database: Scopus
Platform: N/A
Date Searched: 2023-06-08**

(TITLE-ABS-KEY(smartphone* OR "smart phone*" OR cellphone* OR "cell phone*" OR "cellular phone*" OR "mobile phone*" OR app OR apps OR "mobile health" OR mhealth OR "mobile-based" OR "phone-based") OR TITLE-ABS-KEY ( mobile W/1 application* ) OR TITLE-ABS-KEY ( "portable electronic" W/1 application* ) OR TITLE-ABS-KEY ( "portable software" W/1 application* )) and (TITLE-ABS-KEY ( hand W/6 dexterity ) OR TITLE-ABS-KEY ( hand W/6 dexterous*) OR TITLE-ABS-KEY ( hand W/6 grip ) OR TITLE-ABS-KEY ( hand W/6 gripping) OR TITLE-ABS-KEY ( hand W/6 grasp ) OR TITLE-ABS-KEY ( hand W/6 grasping ) OR TITLE-ABS-KEY ( hand W/6 tap ) OR TITLE-ABS-KEY ( hand W/6 tapping ) OR TITLE-ABS-KEY ( hand W/6 strength*) OR TITLE-ABS-KEY ( hand W/6 flex* ) OR TITLE-ABS-KEY ( hand W/6 extent* ) OR TITLE-ABS-KEY ( hand W/6 extend* ) OR TITLE-ABS-KEY ( hand W/6 movement*) OR TITLE-ABS-KEY ( hand W/6 angle ) OR TITLE-ABS-KEY ( hand W/6 angles) OR TITLE-ABS-KEY ( hand W/6 pinch ) OR TITLE-ABS-KEY ( hand W/6 assess*) OR TITLE-ABS-KEY ( hand W/6 function* ) OR TITLE-ABS-KEY ( hand W/6 therap* ) OR TITLE-ABS-KEY ( hand W/6 rehab* ) OR TITLE-ABS-KEY ( hand W/6 analy* ) OR TITLE-ABS-KEY ( hand W/6 "motor skill*" ) OR TITLE-ABS-KEY ( hand W/6 measur* ) OR TITLE-ABS-KEY ( hand W/6 detect* ) OR TITLE-ABS-KEY ( hands W/6 dexterity ) OR TITLE-ABS-KEY ( hands W/6 dexterous*) OR TITLE-ABS-KEY ( hands W/6 grip ) OR TITLE-ABS-KEY ( hands W/6 gripping) OR TITLE-ABS-KEY ( hands W/6 grasp ) OR TITLE-ABS-KEY ( hands W/6 grasping ) OR TITLE-ABS-KEY ( hands W/6 tap ) OR TITLE-ABS-KEY ( hands W/6 tapping ) OR TITLE-ABS-KEY ( hands W/6 strength*) OR TITLE-ABS-KEY ( hands W/6 flex* ) OR TITLE-ABS-KEY ( hands W/6 extent* ) OR TITLE-ABS-KEY ( hands W/6 extend* ) OR TITLE-ABS-KEY ( hands W/6 movement*) OR TITLE-ABS-KEY ( hands W/6 angle ) OR TITLE-ABS-KEY ( hands W/6 angles) OR TITLE-ABS-KEY ( hands W/6 pinch ) OR TITLE-ABS-KEY ( hands W/6 assess*) OR TITLE-ABS-KEY ( hands W/6 function* ) OR TITLE-ABS-KEY ( hands W/6 therap* ) OR TITLE-ABS-KEY ( hands W/6 rehab* ) OR TITLE-ABS-KEY ( hands W/6 analy* ) OR TITLE-ABS-KEY ( hands W/6 "motor skill*" ) OR TITLE-ABS-KEY ( hands W/6 measur* ) OR TITLE-ABS-KEY ( hands W/6 detect* ) OR TITLE-ABS-KEY ( handgrip* W/6 dexterity ) OR TITLE-ABS-KEY ( handgrip* W/6 dexterous*) OR TITLE-ABS-KEY ( handgrip* W/6 grip ) OR TITLE-ABS-KEY ( handgrip* W/6 gripping) OR TITLE-ABS-KEY ( handgrip* W/6 grasp ) OR TITLE-ABS-KEY ( handgrip* W/6 grasping ) OR TITLE-ABS-KEY ( handgrip* W/6 tap ) OR TITLE-ABS-KEY ( handgrip* W/6 tapping ) OR TITLE-ABS-KEY ( handgrip* W/6 strength*) OR TITLE-ABS-KEY ( handgrip* W/6 flex* ) OR TITLE-ABS-KEY ( handgrip* W/6 extent* ) OR TITLE-ABS-KEY ( handgrip* W/6 extend* ) OR TITLE-ABS-KEY ( handgrip* W/6 movement*) OR TITLE-ABS-KEY ( handgrip* W/6 angle ) OR TITLE-ABS-KEY ( handgrip* W/6 angles) OR TITLE-ABS-KEY ( handgrip* W/6 pinch ) OR TITLE-ABS-KEY ( handgrip* W/6 assess*) OR TITLE-ABS-KEY ( handgrip* W/6 function* ) OR TITLE-ABS-KEY ( handgrip* W/6 therap* ) OR TITLE-ABS-KEY ( handgrip* W/6 rehab* ) OR TITLE-ABS-KEY ( handgrip* W/6 analy* ) OR TITLE-ABS-KEY ( handgrip* W/6 "motor skill*" ) OR TITLE-ABS-KEY ( handgrip* W/6 measur* ) OR TITLE-ABS-KEY ( handgrip* W/6 detect* ) OR TITLE-ABS-KEY ( finger* W/6 dexterity ) OR TITLE-ABS-KEY ( finger* W/6 dexterous*) OR TITLE-ABS-KEY ( finger* W/6 grip ) OR TITLE-ABS-KEY ( finger* W/6 gripping) OR TITLE-ABS-KEY ( finger* W/6 grasp ) OR TITLE-ABS-KEY ( finger* W/6 grasping ) OR TITLE-ABS-KEY ( finger* W/6 tap ) OR TITLE-ABS-KEY ( finger* W/6 tapping ) OR TITLE-ABS-KEY ( finger* W/6 strength*) OR TITLE-ABS-KEY ( finger* W/6 flex* ) OR TITLE-ABS-KEY ( finger* W/6 extent* ) OR TITLE-ABS-KEY ( finger* W/6 extend* ) OR TITLE-ABS-KEY ( finger* W/6 movement*) OR TITLE-ABS-KEY ( finger* W/6 angle ) OR TITLE-ABS-KEY ( finger* W/6 angles) OR TITLE-ABS-KEY ( finger* W/6 pinch ) OR TITLE-ABS-KEY ( finger* W/6 assess*) OR TITLE-ABS-KEY ( finger* W/6 function* ) OR TITLE-ABS-KEY ( finger* W/6 therap* ) OR TITLE-ABS-KEY ( finger* W/6 rehab* ) OR TITLE-ABS-KEY ( finger* W/6 analy* ) OR TITLE-ABS-KEY ( finger* W/6 "motor skill*" ) OR TITLE-ABS-KEY ( finger* W/6 measur* ) OR TITLE-ABS-KEY ( finger* W/6 detect* ) OR TITLE-ABS-KEY ( thumb* W/6 dexterity ) OR TITLE-ABS-KEY ( thumb* W/6 dexterous*) OR TITLE-ABS-KEY ( thumb* W/6 grip ) OR TITLE-ABS-KEY ( thumb* W/6 gripping) OR TITLE-ABS-KEY ( thumb* W/6 grasp ) OR TITLE-ABS-KEY ( thumb* W/6 grasping ) OR TITLE-ABS-KEY ( thumb* W/6 tap ) OR TITLE-ABS-KEY ( thumb* W/6 tapping ) OR TITLE-ABS-KEY ( thumb* W/6 strength*) OR TITLE-ABS-KEY ( thumb* W/6 flex* ) OR TITLE-ABS-KEY ( thumb* W/6 extent* ) OR TITLE-ABS-KEY ( thumb* W/6 extend* ) OR TITLE-ABS-KEY ( thumb* W/6 movement*) OR TITLE-ABS-KEY ( thumb* W/6 angle ) OR TITLE-ABS-KEY ( thumb* W/6 angles) OR TITLE-ABS-KEY ( thumb* W/6 pinch ) OR TITLE-ABS-KEY ( thumb* W/6 assess*) OR TITLE-ABS-KEY ( thumb* W/6 function* ) OR TITLE-ABS-KEY ( thumb* W/6 therap* ) OR TITLE-ABS-KEY ( thumb* W/6 rehab* ) OR TITLE-ABS-KEY ( thumb* W/6 analy* ) OR TITLE-ABS-KEY ( thumb* W/6 "motor skill*" ) OR TITLE-ABS-KEY ( thumb* W/6 measur* ) OR TITLE-ABS-KEY ( thumb* W/6 detect* ) OR TITLE-ABS-KEY ( forefinger* W/6 dexterity ) OR TITLE-ABS-KEY ( forefinger* W/6 dexterous*) OR TITLE-ABS-KEY ( forefinger* W/6 grip ) OR TITLE-ABS-KEY ( forefinger* W/6 gripping) OR TITLE-ABS-KEY ( forefinger* W/6 grasp ) OR TITLE-ABS-KEY ( forefinger* W/6 grasping ) OR TITLE-ABS-KEY ( forefinger* W/6 tap ) OR TITLE-ABS-KEY ( forefinger* W/6 tapping ) OR TITLE-ABS-KEY ( forefinger* W/6 strength*) OR TITLE-ABS-KEY ( forefinger* W/6 flex* ) OR TITLE-ABS-KEY ( forefinger* W/6 extent* ) OR TITLE-ABS-KEY ( forefinger* W/6 extend* ) OR TITLE-ABS-KEY ( forefinger* W/6 movement*) OR TITLE-ABS-KEY ( forefinger* W/6 angle ) OR TITLE-ABS-KEY ( forefinger* W/6 angles) OR TITLE-ABS-KEY ( forefinger* W/6 pinch ) OR TITLE-ABS-KEY ( forefinger* W/6 assess*) OR TITLE-ABS-KEY ( forefinger* W/6 function* ) OR TITLE-ABS-KEY ( forefinger* W/6 therap* ) OR TITLE-ABS-KEY ( forefinger* W/6 rehab* ) OR TITLE-ABS-KEY ( forefinger* W/6 analy* ) OR TITLE-ABS-KEY ( forefinger* W/6 "motor skill*" ) OR TITLE-ABS-KEY ( forefinger* W/6 measur* ) OR TITLE-ABS-KEY ( forefinger* W/6 detect* ) OR TITLE-ABS-KEY ( pinky W/6 dexterity ) OR TITLE-ABS-KEY ( pinky W/6 dexterous*) OR TITLE-ABS-KEY ( pinky W/6 grip ) OR TITLE-ABS-KEY ( pinky W/6 gripping) OR TITLE-ABS-KEY ( pinky W/6 grasp ) OR TITLE-ABS-KEY ( pinky W/6 grasping ) OR TITLE-ABS-KEY ( pinky W/6 tap ) OR TITLE-ABS-KEY ( pinky W/6 tapping ) OR TITLE-ABS-KEY ( pinky W/6 strength*) OR TITLE-ABS-KEY ( pinky W/6 flex* ) OR TITLE-ABS-KEY ( pinky W/6 extent* ) OR TITLE-ABS-KEY ( pinky W/6 extend* ) OR TITLE-ABS-KEY ( pinky W/6 movement*) OR TITLE-ABS-KEY ( pinky W/6 angle ) OR TITLE-ABS-KEY ( pinky W/6 angles) OR TITLE-ABS-KEY ( pinky W/6 pinch ) OR TITLE-ABS-KEY ( pinky W/6 assess*) OR TITLE-ABS-KEY ( pinky W/6 function* ) OR TITLE-ABS-KEY ( pinky W/6 therap* ) OR TITLE-ABS-KEY ( pinky W/6 rehab* ) OR TITLE-ABS-KEY ( pinky W/6 analy* ) OR TITLE-ABS-KEY ( pinky W/6 "motor skill*" ) OR TITLE-ABS-KEY ( pinky W/6 measur* ) OR TITLE-ABS-KEY ( pinky W/6 detect* ) OR TITLE-ABS-KEY ( pinkies W/6 dexterity ) OR TITLE-ABS-KEY ( pinkies W/6 dexterous*) OR TITLE-ABS-KEY ( pinkies W/6 grip ) OR TITLE-ABS-KEY ( pinkies W/6 gripping) OR TITLE-ABS-KEY ( pinkies W/6 grasp ) OR TITLE-ABS-KEY ( pinkies W/6 grasping ) OR TITLE-ABS-KEY ( pinkies W/6 tap ) OR TITLE-ABS-KEY ( pinkies W/6 tapping ) OR TITLE-ABS-KEY ( pinkies W/6 strength*) OR TITLE-ABS-KEY ( pinkies W/6 flex* ) OR TITLE-ABS-KEY ( pinkies W/6 extent* ) OR TITLE-ABS-KEY ( pinkies W/6 extend* ) OR TITLE-ABS-KEY ( pinkies W/6 movement*) OR TITLE-ABS-KEY ( pinkies W/6 angle ) OR TITLE-ABS-KEY ( pinkies W/6 angles) OR TITLE-ABS-KEY ( pinkies W/6 pinch ) OR TITLE-ABS-KEY ( pinkies W/6 assess*) OR TITLE-ABS-KEY ( pinkies W/6 function* ) OR TITLE-ABS-KEY ( pinkies W/6 therap* ) OR TITLE-ABS-KEY ( pinkies W/6 rehab* ) OR TITLE-ABS-KEY ( pinkies W/6 analy* ) OR TITLE-ABS-KEY ( pinkies W/6 "motor skill*" ) OR TITLE-ABS-KEY ( pinkies W/6 measur* ) OR TITLE-ABS-KEY ( pinkies W/6 detect* ) OR TITLE-ABS-KEY ( metacarpus W/6 dexterity ) OR TITLE-ABS-KEY ( metacarpus W/6 dexterous*) OR TITLE-ABS-KEY ( metacarpus W/6 grip ) OR TITLE-ABS-KEY ( metacarpus W/6 gripping) OR TITLE-ABS-KEY ( metacarpus W/6 grasp ) OR TITLE-ABS-KEY ( metacarpus W/6 grasping ) OR TITLE-ABS-KEY ( metacarpus W/6 tap ) OR TITLE-ABS-KEY ( metacarpus W/6 tapping ) OR TITLE-ABS-KEY ( metacarpus W/6 strength*) OR TITLE-ABS-KEY ( metacarpus W/6 flex* ) OR TITLE-ABS-KEY ( metacarpus W/6 extent* ) OR TITLE-ABS-KEY ( metacarpus W/6 extend* ) OR TITLE-ABS-KEY ( metacarpus W/6 movement*) OR TITLE-ABS-KEY ( metacarpus W/6 angle ) OR TITLE-ABS-KEY ( metacarpus W/6 angles) OR TITLE-ABS-KEY ( metacarpus W/6 pinch ) OR TITLE-ABS-KEY ( metacarpus W/6 assess*) OR TITLE-ABS-KEY ( metacarpus W/6 function* ) OR TITLE-ABS-KEY ( metacarpus W/6 therap* ) OR TITLE-ABS-KEY ( metacarpus W/6 rehab* ) OR TITLE-ABS-KEY ( metacarpus W/6 analy* ) OR TITLE-ABS-KEY ( metacarpus W/6 "motor skill*" ) OR TITLE-ABS-KEY ( metacarpus W/6 measur* ) OR TITLE-ABS-KEY ( metacarpus W/6 detect* ) OR TITLE-ABS-KEY ( metacarpal* W/6 dexterity ) OR TITLE-ABS-KEY ( metacarpal* W/6 dexterous*) OR TITLE-ABS-KEY ( metacarpal* W/6 grip ) OR TITLE-ABS-KEY ( metacarpal* W/6 gripping) OR TITLE-ABS-KEY ( metacarpal* W/6 grasp ) OR TITLE-ABS-KEY ( metacarpal* W/6 grasping ) OR TITLE-ABS-KEY ( metacarpal* W/6 tap ) OR TITLE-ABS-KEY ( metacarpal* W/6 tapping ) OR TITLE-ABS-KEY ( metacarpal* W/6 strength*) OR TITLE-ABS-KEY ( metacarpal* W/6 flex* ) OR TITLE-ABS-KEY ( metacarpal* W/6 extent* ) OR TITLE-ABS-KEY ( metacarpal* W/6 extend* ) OR TITLE-ABS-KEY ( metacarpal* W/6 movement*) OR TITLE-ABS-KEY ( metacarpal* W/6 angle ) OR TITLE-ABS-KEY ( metacarpal* W/6 angles) OR TITLE-ABS-KEY ( metacarpal* W/6 pinch ) OR TITLE-ABS-KEY ( metacarpal* W/6 assess*) OR TITLE-ABS-KEY ( metacarpal* W/6 function* ) OR TITLE-ABS-KEY ( metacarpal* W/6 therap* ) OR TITLE-ABS-KEY ( metacarpal* W/6 rehab* ) OR TITLE-ABS-KEY ( metacarpal* W/6 analy* ) OR TITLE-ABS-KEY ( metacarpal* W/6 "motor skill*" ) OR TITLE-ABS-KEY ( metacarpal* W/6 measur* ) OR TITLE-ABS-KEY ( metacarpal* W/6 detect* )) AND ( LIMIT-TO ( DOCTYPE,"ar" ) OR LIMIT-TO ( DOCTYPE,"cp" ) ) AND ( LIMIT-TO ( LANGUAGE,"English" ) )

**Database: Compendex
Platform: Engineering Village
Date Searched: 2023-06-07**

(((((( (((((({Cellular telephones} WN CV) OR ({Smartphones} WN CV) OR ({Application programs} WN CV) OR ({iOS (operating system)} WN CV) OR ({Android (operating system)} WN CV) OR ({mHealth} WN CV))))) OR ((((smartphone* OR {smart phone*} OR cellphone* OR {cell phone*} OR {cellular phone*} OR {mobile phone*} OR $app OR $apps OR {mobile health} OR $mhealth OR {mobile-based} OR {phone-based}) WN KY))) OR (((($mobile NEAR/1 $application) WN KY) OR (($portable $electronic NEAR/1 $application) WN KY) OR (($portable $software NEAR/1 $application) WN KY))))) AND ( ((((($hand NEAR/6 $dexterity) WN KY) OR (($hand NEAR/6 $dexterous) WN KY) OR (($hand NEAR/6 $grip) WN KY) OR (($hand NEAR/6 $gripping) WN KY) OR (($hand NEAR/6 $grasp) WN KY) OR (($hand NEAR/6 $grasping) WN KY) OR (($hand NEAR/6 $tap) WN KY) OR (($hand NEAR/6 $tapping) WN KY) OR (($hand NEAR/6 $strength) WN KY) OR (($hand NEAR/6 $flex) WN KY) OR (($hand NEAR/6 $extention) WN KY) OR (($hand NEAR/6 $extend) WN KY) OR (($hand NEAR/6 $movement) WN KY) OR (($hand NEAR/6 $angle) WN KY) OR (($hand NEAR/6 $angles) WN KY) OR (($hand NEAR/6 $pinch) WN KY) OR (($hand NEAR/6 $assess) WN KY) OR (($hand NEAR/6 $function) WN KY) OR (($hand NEAR/6 $therapy) WN KY) OR (($hand NEAR/6 $rehab) WN KY) OR (($hand NEAR/6 $analysis) WN KY) OR (($hand NEAR/6 $motor) WN KY) OR (($hand NEAR/6 $measurement) WN KY) OR (($hand NEAR/6 $detection) WN KY))) OR (((($handgrip NEAR/6 $dexterity) WN KY) OR (($handgrip NEAR/6 $dexterous) WN KY) OR (($handgrip NEAR/6 $grip) WN KY) OR (($handgrip NEAR/6 $gripping) WN KY) OR (($handgrip NEAR/6 $grasp) WN KY) OR (($handgrip NEAR/6 $grasping) WN KY) OR (($handgrip NEAR/6 $tap) WN KY) OR (($handgrip NEAR/6 $tapping) WN KY) OR (($handgrip NEAR/6 $strength) WN KY) OR (($handgrip NEAR/6 $flex) WN KY) OR (($handgrip NEAR/6 $extention) WN KY) OR (($handgrip NEAR/6 $extend) WN KY) OR (($handgrip NEAR/6 $movement) WN KY) OR (($handgrip NEAR/6 $angle) WN KY) OR (($handgrip NEAR/6 $angles) WN KY) OR (($handgrip NEAR/6 $pinch) WN KY) OR (($handgrip NEAR/6 $assess) WN KY) OR (($handgrip NEAR/6 $function) WN KY) OR (($handgrip NEAR/6 $therapy) WN KY) OR (($handgrip NEAR/6 $rehab) WN KY) OR (($handgrip NEAR/6 $analysis) WN KY) OR (($handgrip NEAR/6 $motor) WN KY) OR (($handgrip NEAR/6 $measurement) WN KY) OR (($handgrip NEAR/6 $detection) WN KY))) OR (((($handgrip NEAR/6 $dexterity) WN KY) OR (($handgrip NEAR/6 $dexterous) WN KY) OR (($handgrip NEAR/6 $grip) WN KY) OR (($handgrip NEAR/6 $gripping) WN KY) OR (($handgrip NEAR/6 $grasp) WN KY) OR (($handgrip NEAR/6 $grasping) WN KY) OR (($handgrip NEAR/6 $tap) WN KY) OR (($handgrip NEAR/6 $tapping) WN KY) OR (($handgrip NEAR/6 $strength) WN KY) OR (($handgrip NEAR/6 $flex) WN KY) OR (($handgrip NEAR/6 $extention) WN KY) OR (($handgrip NEAR/6 $extend) WN KY) OR (($handgrip NEAR/6 $movement) WN KY) OR (($handgrip NEAR/6 $angle) WN KY) OR (($handgrip NEAR/6 $angles) WN KY) OR (($handgrip NEAR/6 $pinch) WN KY) OR (($handgrip NEAR/6 $assess) WN KY) OR (($handgrip NEAR/6 $function) WN KY) OR (($handgrip NEAR/6 $therapy) WN KY) OR (($handgrip NEAR/6 $rehab) WN KY) OR (($handgrip NEAR/6 $analysis) WN KY) OR (($handgrip NEAR/6 $motor) WN KY) OR (($handgrip NEAR/6 $measurement) WN KY) OR (($handgrip NEAR/6 $detection) WN KY) OR (($hand NEAR/6 $dexterity) WN KY) OR (($hand NEAR/6 $dexterous) WN KY) OR (($hand NEAR/6 $grip) WN KY) OR (($hand NEAR/6 $gripping) WN KY) OR (($hand NEAR/6 $grasp) WN KY) OR (($hand NEAR/6 $grasping) WN KY) OR (($hand NEAR/6 $tap) WN KY) OR (($hand NEAR/6 $tapping) WN KY) OR (($hand NEAR/6 $strength) WN KY) OR (($hand NEAR/6 $flex) WN KY) OR (($hand NEAR/6 $extention) WN KY) OR (($hand NEAR/6 $extend) WN KY) OR (($hand NEAR/6 $movement) WN KY) OR (($hand NEAR/6 $angle) WN KY) OR (($hand NEAR/6 $angles) WN KY) OR (($hand NEAR/6 $pinch) WN KY) OR (($hand NEAR/6 $assess) WN KY) OR (($hand NEAR/6 $function) WN KY) OR (($hand NEAR/6 $therapy) WN KY) OR (($hand NEAR/6 $rehab) WN KY) OR (($hand NEAR/6 $analysis) WN KY) OR (($hand NEAR/6 $motor) WN KY) OR (($hand NEAR/6 $measurement) WN KY) OR (($hand NEAR/6 $detection) WN KY))) OR (((($finger NEAR/6 $dexterity) WN KY) OR (($finger NEAR/6 $dexterous) WN KY) OR (($finger NEAR/6 $grip) WN KY) OR (($finger NEAR/6 $gripping) WN KY) OR (($finger NEAR/6 $grasp) WN KY) OR (($finger NEAR/6 $grasping) WN KY) OR (($finger NEAR/6 $tap) WN KY) OR (($finger NEAR/6 $tapping) WN KY) OR (($finger NEAR/6 $strength) WN KY) OR (($finger NEAR/6 $flex) WN KY) OR (($finger NEAR/6 $extention) WN KY) OR (($finger NEAR/6 $extend) WN KY) OR (($finger NEAR/6 $movement) WN KY) OR (($finger NEAR/6 $angle) WN KY) OR (($finger NEAR/6 $angles) WN KY) OR (($finger NEAR/6 $pinch) WN KY) OR (($finger NEAR/6 $assess) WN KY) OR (($finger NEAR/6 $function) WN KY) OR (($finger NEAR/6 $therapy) WN KY) OR (($finger NEAR/6 $rehab) WN KY) OR (($finger NEAR/6 $analysis) WN KY) OR (($finger NEAR/6 $motor) WN KY) OR (($finger NEAR/6 $measurement) WN KY) OR (($finger NEAR/6 $detection) WN KY))) OR (((($thumb NEAR/6 $dexterity) WN KY) OR (($thumb NEAR/6 $dexterous) WN KY) OR (($thumb NEAR/6 $grip) WN KY) OR (($thumb NEAR/6 $gripping) WN KY) OR (($thumb NEAR/6 $grasp) WN KY) OR (($thumb NEAR/6 $grasping) WN KY) OR (($thumb NEAR/6 $tap) WN KY) OR (($thumb NEAR/6 $tapping) WN KY) OR (($thumb NEAR/6 $strength) WN KY) OR (($thumb NEAR/6 $flex) WN KY) OR (($thumb NEAR/6 $extention) WN KY) OR (($thumb NEAR/6 $extend) WN KY) OR (($thumb NEAR/6 $movement) WN KY) OR (($thumb NEAR/6 $angle) WN KY) OR (($thumb NEAR/6 $angles) WN KY) OR (($thumb NEAR/6 $pinch) WN KY) OR (($thumb NEAR/6 $assess) WN KY) OR (($thumb NEAR/6 $function) WN KY) OR (($thumb NEAR/6 $therapy) WN KY) OR (($thumb NEAR/6 $rehab) WN KY) OR (($thumb NEAR/6 $analysis) WN KY) OR (($thumb NEAR/6 $motor) WN KY) OR (($thumb NEAR/6 $measurement) WN KY) OR (($thumb NEAR/6 $detection) WN KY))) OR (((($forefinger NEAR/6 $dexterity) WN KY) OR (($forefinger NEAR/6 $dexterous) WN KY) OR (($forefinger NEAR/6 $grip) WN KY) OR (($forefinger NEAR/6 $gripping) WN KY) OR (($forefinger NEAR/6 $grasp) WN KY) OR (($forefinger NEAR/6 $grasping) WN KY) OR (($forefinger NEAR/6 $tap) WN KY) OR (($forefinger NEAR/6 $tapping) WN KY) OR (($forefinger NEAR/6 $strength) WN KY) OR (($forefinger NEAR/6 $flex) WN KY) OR (($forefinger NEAR/6 $extention) WN KY) OR (($forefinger NEAR/6 $extend) WN KY) OR (($forefinger NEAR/6 $movement) WN KY) OR (($forefinger NEAR/6 $angle) WN KY) OR (($forefinger NEAR/6 $angles) WN KY) OR (($forefinger NEAR/6 $pinch) WN KY) OR (($forefinger NEAR/6 $assess) WN KY) OR (($forefinger NEAR/6 $function) WN KY) OR (($forefinger NEAR/6 $therapy) WN KY) OR (($forefinger NEAR/6 $rehab) WN KY) OR (($forefinger NEAR/6 $analysis) WN KY) OR (($forefinger NEAR/6 $motor) WN KY) OR (($forefinger NEAR/6 $measurement) WN KY) OR (($forefinger NEAR/6 $detection) WN KY))) OR (((($pinky NEAR/6 $dexterity) WN KY) OR (($pinky NEAR/6 $dexterous) WN KY) OR (($pinky NEAR/6 $grip) WN KY) OR (($pinky NEAR/6 $gripping) WN KY) OR (($pinky NEAR/6 $grasp) WN KY) OR (($pinky NEAR/6 $grasping) WN KY) OR (($pinky NEAR/6 $tap) WN KY) OR (($pinky NEAR/6 $tapping) WN KY) OR (($pinky NEAR/6 $strength) WN KY) OR (($pinky NEAR/6 $flex) WN KY) OR (($pinky NEAR/6 $extention) WN KY) OR (($pinky NEAR/6 $extend) WN KY) OR (($pinky NEAR/6 $movement) WN KY) OR (($pinky NEAR/6 $angle) WN KY) OR (($pinky NEAR/6 $angles) WN KY) OR (($pinky NEAR/6 $pinch) WN KY) OR (($pinky NEAR/6 $assess) WN KY) OR (($pinky NEAR/6 $function) WN KY) OR (($pinky NEAR/6 $therapy) WN KY) OR (($pinky NEAR/6 $rehab) WN KY) OR (($pinky NEAR/6 $analysis) WN KY) OR (($pinky NEAR/6 $motor) WN KY) OR (($pinky NEAR/6 $measurement) WN KY) OR (($pinky NEAR/6 $detection) WN KY))) OR (((($metacarpus NEAR/6 $dexterity) WN KY) OR (($metacarpus NEAR/6 $dexterous) WN KY) OR (($metacarpus NEAR/6 $grip) WN KY) OR (($metacarpus NEAR/6 $gripping) WN KY) OR (($metacarpus NEAR/6 $grasp) WN KY) OR (($metacarpus NEAR/6 $grasping) WN KY) OR (($metacarpus NEAR/6 $tap) WN KY) OR (($metacarpus NEAR/6 $tapping) WN KY) OR (($metacarpus NEAR/6 $strength) WN KY) OR (($metacarpus NEAR/6 $flex) WN KY) OR (($metacarpus NEAR/6 $extention) WN KY) OR (($metacarpus NEAR/6 $extend) WN KY) OR (($metacarpus NEAR/6 $movement) WN KY) OR (($metacarpus NEAR/6 $angle) WN KY) OR (($metacarpus NEAR/6 $angles) WN KY) OR (($metacarpus NEAR/6 $pinch) WN KY) OR (($metacarpus NEAR/6 $assess) WN KY) OR (($metacarpus NEAR/6 $function) WN KY) OR (($metacarpus NEAR/6 $therapy) WN KY) OR (($metacarpus NEAR/6 $rehab) WN KY) OR (($metacarpus NEAR/6 $analysis) WN KY) OR (($metacarpus NEAR/6 $motor) WN KY) OR (($metacarpus NEAR/6 $measurement) WN KY) OR (($metacarpus NEAR/6 $detection) WN KY))))))) AND (({ca} OR {ja}) WN DT)) AND ({english} WN LA)))

**Database: INSPEC
Platform: Engineering Village
Date Searched: 2023-06-07**

(((((( (((((($hand NEAR/6 $dexterity) WN KY) OR (($hand NEAR/6 $dexterous) WN KY) OR (($hand NEAR/6 $grip) WN KY) OR (($hand NEAR/6 $gripping) WN KY) OR (($hand NEAR/6 $grasp) WN KY) OR (($hand NEAR/6 $grasping) WN KY) OR (($hand NEAR/6 $tap) WN KY) OR (($hand NEAR/6 $tapping) WN KY) OR (($hand NEAR/6 $strength) WN KY) OR (($hand NEAR/6 $flex) WN KY) OR (($hand NEAR/6 $extention) WN KY) OR (($hand NEAR/6 $extend) WN KY) OR (($hand NEAR/6 $movement) WN KY) OR (($hand NEAR/6 $angle) WN KY) OR (($hand NEAR/6 $angles) WN KY) OR (($hand NEAR/6 $pinch) WN KY) OR (($hand NEAR/6 $assess) WN KY) OR (($hand NEAR/6 $function) WN KY) OR (($hand NEAR/6 $therapy) WN KY) OR (($hand NEAR/6 $rehab) WN KY) OR (($hand NEAR/6 $analysis) WN KY) OR (($hand NEAR/6 $motor) WN KY) OR (($hand NEAR/6 $measurement) WN KY) OR (($hand NEAR/6 $detection) WN KY))) OR (((($handgrip NEAR/6 $dexterity) WN KY) OR (($handgrip NEAR/6 $dexterous) WN KY) OR (($handgrip NEAR/6 $grip) WN KY) OR (($handgrip NEAR/6 $gripping) WN KY) OR (($handgrip NEAR/6 $grasp) WN KY) OR (($handgrip NEAR/6 $grasping) WN KY) OR (($handgrip NEAR/6 $tap) WN KY) OR (($handgrip NEAR/6 $tapping) WN KY) OR (($handgrip NEAR/6 $strength) WN KY) OR (($handgrip NEAR/6 $flex) WN KY) OR (($handgrip NEAR/6 $extention) WN KY) OR (($handgrip NEAR/6 $extend) WN KY) OR (($handgrip NEAR/6 $movement) WN KY) OR (($handgrip NEAR/6 $angle) WN KY) OR (($handgrip NEAR/6 $angles) WN KY) OR (($handgrip NEAR/6 $pinch) WN KY) OR (($handgrip NEAR/6 $assess) WN KY) OR (($handgrip NEAR/6 $function) WN KY) OR (($handgrip NEAR/6 $therapy) WN KY) OR (($handgrip NEAR/6 $rehab) WN KY) OR (($handgrip NEAR/6 $analysis) WN KY) OR (($handgrip NEAR/6 $motor) WN KY) OR (($handgrip NEAR/6 $measurement) WN KY) OR (($handgrip NEAR/6 $detection) WN KY))) OR (((($handgrip NEAR/6 $dexterity) WN KY) OR (($handgrip NEAR/6 $dexterous) WN KY) OR (($handgrip NEAR/6 $grip) WN KY) OR (($handgrip NEAR/6 $gripping) WN KY) OR (($handgrip NEAR/6 $grasp) WN KY) OR (($handgrip NEAR/6 $grasping) WN KY) OR (($handgrip NEAR/6 $tap) WN KY) OR (($handgrip NEAR/6 $tapping) WN KY) OR (($handgrip NEAR/6 $strength) WN KY) OR (($handgrip NEAR/6 $flex) WN KY) OR (($handgrip NEAR/6 $extention) WN KY) OR (($handgrip NEAR/6 $extend) WN KY) OR (($handgrip NEAR/6 $movement) WN KY) OR (($handgrip NEAR/6 $angle) WN KY) OR (($handgrip NEAR/6 $angles) WN KY) OR (($handgrip NEAR/6 $pinch) WN KY) OR (($handgrip NEAR/6 $assess) WN KY) OR (($handgrip NEAR/6 $function) WN KY) OR (($handgrip NEAR/6 $therapy) WN KY) OR (($handgrip NEAR/6 $rehab) WN KY) OR (($handgrip NEAR/6 $analysis) WN KY) OR (($handgrip NEAR/6 $motor) WN KY) OR (($handgrip NEAR/6 $measurement) WN KY) OR (($handgrip NEAR/6 $detection) WN KY) OR (($hand NEAR/6 $dexterity) WN KY) OR (($hand NEAR/6 $dexterous) WN KY) OR (($hand NEAR/6 $grip) WN KY) OR (($hand NEAR/6 $gripping) WN KY) OR (($hand NEAR/6 $grasp) WN KY) OR (($hand NEAR/6 $grasping) WN KY) OR (($hand NEAR/6 $tap) WN KY) OR (($hand NEAR/6 $tapping) WN KY) OR (($hand NEAR/6 $strength) WN KY) OR (($hand NEAR/6 $flex) WN KY) OR (($hand NEAR/6 $extention) WN KY) OR (($hand NEAR/6 $extend) WN KY) OR (($hand NEAR/6 $movement) WN KY) OR (($hand NEAR/6 $angle) WN KY) OR (($hand NEAR/6 $angles) WN KY) OR (($hand NEAR/6 $pinch) WN KY) OR (($hand NEAR/6 $assess) WN KY) OR (($hand NEAR/6 $function) WN KY) OR (($hand NEAR/6 $therapy) WN KY) OR (($hand NEAR/6 $rehab) WN KY) OR (($hand NEAR/6 $analysis) WN KY) OR (($hand NEAR/6 $motor) WN KY) OR (($hand NEAR/6 $measurement) WN KY) OR (($hand NEAR/6 $detection) WN KY))) OR (((($finger NEAR/6 $dexterity) WN KY) OR (($finger NEAR/6 $dexterous) WN KY) OR (($finger NEAR/6 $grip) WN KY) OR (($finger NEAR/6 $gripping) WN KY) OR (($finger NEAR/6 $grasp) WN KY) OR (($finger NEAR/6 $grasping) WN KY) OR (($finger NEAR/6 $tap) WN KY) OR (($finger NEAR/6 $tapping) WN KY) OR (($finger NEAR/6 $strength) WN KY) OR (($finger NEAR/6 $flex) WN KY) OR (($finger NEAR/6 $extention) WN KY) OR (($finger NEAR/6 $extend) WN KY) OR (($finger NEAR/6 $movement) WN KY) OR (($finger NEAR/6 $angle) WN KY) OR (($finger NEAR/6 $angles) WN KY) OR (($finger NEAR/6 $pinch) WN KY) OR (($finger NEAR/6 $assess) WN KY) OR (($finger NEAR/6 $function) WN KY) OR (($finger NEAR/6 $therapy) WN KY) OR (($finger NEAR/6 $rehab) WN KY) OR (($finger NEAR/6 $analysis) WN KY) OR (($finger NEAR/6 $motor) WN KY) OR (($finger NEAR/6 $measurement) WN KY) OR (($finger NEAR/6 $detection) WN KY))) OR (((($thumb NEAR/6 $dexterity) WN KY) OR (($thumb NEAR/6 $dexterous) WN KY) OR (($thumb NEAR/6 $grip) WN KY) OR (($thumb NEAR/6 $gripping) WN KY) OR (($thumb NEAR/6 $grasp) WN KY) OR (($thumb NEAR/6 $grasping) WN KY) OR (($thumb NEAR/6 $tap) WN KY) OR (($thumb NEAR/6 $tapping) WN KY) OR (($thumb NEAR/6 $strength) WN KY) OR (($thumb NEAR/6 $flex) WN KY) OR (($thumb NEAR/6 $extention) WN KY) OR (($thumb NEAR/6 $extend) WN KY) OR (($thumb NEAR/6 $movement) WN KY) OR (($thumb NEAR/6 $angle) WN KY) OR (($thumb NEAR/6 $angles) WN KY) OR (($thumb NEAR/6 $pinch) WN KY) OR (($thumb NEAR/6 $assess) WN KY) OR (($thumb NEAR/6 $function) WN KY) OR (($thumb NEAR/6 $therapy) WN KY) OR (($thumb NEAR/6 $rehab) WN KY) OR (($thumb NEAR/6 $analysis) WN KY) OR (($thumb NEAR/6 $motor) WN KY) OR (($thumb NEAR/6 $measurement) WN KY) OR (($thumb NEAR/6 $detection) WN KY))) OR (((($forefinger NEAR/6 $dexterity) WN KY) OR (($forefinger NEAR/6 $dexterous) WN KY) OR (($forefinger NEAR/6 $grip) WN KY) OR (($forefinger NEAR/6 $gripping) WN KY) OR (($forefinger NEAR/6 $grasp) WN KY) OR (($forefinger NEAR/6 $grasping) WN KY) OR (($forefinger NEAR/6 $tap) WN KY) OR (($forefinger NEAR/6 $tapping) WN KY) OR (($forefinger NEAR/6 $strength) WN KY) OR (($forefinger NEAR/6 $flex) WN KY) OR (($forefinger NEAR/6 $extention) WN KY) OR (($forefinger NEAR/6 $extend) WN KY) OR (($forefinger NEAR/6 $movement) WN KY) OR (($forefinger NEAR/6 $angle) WN KY) OR (($forefinger NEAR/6 $angles) WN KY) OR (($forefinger NEAR/6 $pinch) WN KY) OR (($forefinger NEAR/6 $assess) WN KY) OR (($forefinger NEAR/6 $function) WN KY) OR (($forefinger NEAR/6 $therapy) WN KY) OR (($forefinger NEAR/6 $rehab) WN KY) OR (($forefinger NEAR/6 $analysis) WN KY) OR (($forefinger NEAR/6 $motor) WN KY) OR (($forefinger NEAR/6 $measurement) WN KY) OR (($forefinger NEAR/6 $detection) WN KY))) OR (((($pinky NEAR/6 $dexterity) WN KY) OR (($pinky NEAR/6 $dexterous) WN KY) OR (($pinky NEAR/6 $grip) WN KY) OR (($pinky NEAR/6 $gripping) WN KY) OR (($pinky NEAR/6 $grasp) WN KY) OR (($pinky NEAR/6 $grasping) WN KY) OR (($pinky NEAR/6 $tap) WN KY) OR (($pinky NEAR/6 $tapping) WN KY) OR (($pinky NEAR/6 $strength) WN KY) OR (($pinky NEAR/6 $flex) WN KY) OR (($pinky NEAR/6 $extention) WN KY) OR (($pinky NEAR/6 $extend) WN KY) OR (($pinky NEAR/6 $movement) WN KY) OR (($pinky NEAR/6 $angle) WN KY) OR (($pinky NEAR/6 $angles) WN KY) OR (($pinky NEAR/6 $pinch) WN KY) OR (($pinky NEAR/6 $assess) WN KY) OR (($pinky NEAR/6 $function) WN KY) OR (($pinky NEAR/6 $therapy) WN KY) OR (($pinky NEAR/6 $rehab) WN KY) OR (($pinky NEAR/6 $analysis) WN KY) OR (($pinky NEAR/6 $motor) WN KY) OR (($pinky NEAR/6 $measurement) WN KY) OR (($pinky NEAR/6 $detection) WN KY))) OR (((($metacarpus NEAR/6 $dexterity) WN KY) OR (($metacarpus NEAR/6 $dexterous) WN KY) OR (($metacarpus NEAR/6 $grip) WN KY) OR (($metacarpus NEAR/6 $gripping) WN KY) OR (($metacarpus NEAR/6 $grasp) WN KY) OR (($metacarpus NEAR/6 $grasping) WN KY) OR (($metacarpus NEAR/6 $tap) WN KY) OR (($metacarpus NEAR/6 $tapping) WN KY) OR (($metacarpus NEAR/6 $strength) WN KY) OR (($metacarpus NEAR/6 $flex) WN KY) OR (($metacarpus NEAR/6 $extention) WN KY) OR (($metacarpus NEAR/6 $extend) WN KY) OR (($metacarpus NEAR/6 $movement) WN KY) OR (($metacarpus NEAR/6 $angle) WN KY) OR (($metacarpus NEAR/6 $angles) WN KY) OR (($metacarpus NEAR/6 $pinch) WN KY) OR (($metacarpus NEAR/6 $assess) WN KY) OR (($metacarpus NEAR/6 $function) WN KY) OR (($metacarpus NEAR/6 $therapy) WN KY) OR (($metacarpus NEAR/6 $rehab) WN KY) OR (($metacarpus NEAR/6 $analysis) WN KY) OR (($metacarpus NEAR/6 $motor) WN KY) OR (($metacarpus NEAR/6 $measurement) WN KY) OR (($metacarpus NEAR/6 $detection) WN KY)))))) AND ( (((((({smart phones} WN CV) OR ({Android (operating system)} WN CV) OR ({iOS (operating system)} WN CV))))) OR (((((smartphone* OR {smart phone*} OR cellphone* OR {cell phone*} OR {cellular phone*} OR {mobile phone*} OR app OR apps OR {mobile health} OR mhealth OR {mobile-based} OR {phone-based}) WN KY)))) OR (((((mobile NEAR/1 $application) WN KY) OR ((portable electronic NEAR/1 $application) WN KY) OR ((portable software NEAR/1 $application) WN KY)))))))) AND (({ca} OR {ja}) WN DT)) AND ({english} WN LA)))

**Database: ACM Digital Library
Platform: N/A
Date Searched: 2023-06-08**

Title:(smartphone* "smart phone" "smart phones" cellphone* "cell phone" "cell phones" "cellular phone" "cellular phones" "mobile phone" "mobile phones" app apps "mobile health" mhealth "mobile\-based" "phone\-based" "mobile application" "portable electronic application" "postable software application") AND Title:(hand? handgrip? finger? thumb? forefinger? pinky pinkie? metacarpus metacarpal*)

Limits: Research Article

**Database: IEEE Xplore
Platform: N/A
Date Searched: 2023-06-08**

1. (("All Metadata":smartphone OR "All Metadata":"smart phone" OR "All Metadata":cellphone OR "All Metadata":"mobile phone" OR "All Metadata":app OR "All Metadata":"mobile health" OR "All Metadata":mhealth) AND ("All Metadata":hand OR "All Metadata":handstrength OR "All Metadata":finger OR "All Metadata":thumb OR "All Metadata":forefinger OR "All Metadata":pinkie OR "All Metadata":metacarpus OR "All Metadata":metacarpal) AND ("All Metadata":dexterity OR "All Metadata":dexterous* OR "All Metadata":grip* OR "All Metadata":grasp OR "All Metadata":tap OR "All Metadata":strength* OR "All Metadata":flex* OR "All Metadata":extent*) )
   Filters: Conferences, Journals, Early Access Articles
   Results: 288
2. (("All Metadata":smartphone OR "All Metadata":"smart phone" OR "All Metadata":cellphone OR "All Metadata":"mobile phone" OR "All Metadata":app OR "All Metadata":"mobile health" OR "All Metadata":mhealth) AND ("All Metadata":hand OR "All Metadata":handstrength OR "All Metadata":finger OR "All Metadata":thumb OR "All Metadata":forefinger OR "All Metadata":pinkie OR "All Metadata":metacarpus OR "All Metadata":metacarpal) AND ("All Metadata":extend* OR "All Metadata":movement* OR "All Metadata":angle* OR "All Metadata":pinch OR "All Metadata":assess* OR "All Metadata":function* OR "All Metadata":therap* OR "All Metadata":rehab*) )

Filters: Conferences, Journals, Early Access Articles
Results: 762

1. (("All Metadata":smartphone OR "All Metadata":"smart phone" OR "All Metadata":cellphone OR "All Metadata":"mobile phone" OR "All Metadata":app OR "All Metadata":"mobile health" OR "All Metadata":mhealth) AND ("All Metadata":hand OR "All Metadata":handstrength OR "All Metadata":finger OR "All Metadata":thumb OR "All Metadata":forefinger OR "All Metadata":pinkie OR "All Metadata":metacarpus OR "All Metadata":metacarpal) AND ("All Metadata":analys* OR "All Metadata":"motor skill"" OR "All Metadata":measur* OR "All Metadata":detect*) )
   Filters: Conferences, Journals, Early Access Articles
   Result: 1427
